# Photosynthetic efficiency and transcriptome analysis of *Dunaliella salina* under hypersaline: a retrograde signaling mechanism in the chloroplast

**DOI:** 10.3389/fpls.2023.1192258

**Published:** 2023-06-21

**Authors:** Pavithra Ramachandran, Naveen Kumar Pandey, Ranay Mohan Yadav, Praveena Suresh, Aman Kumar, Rajagopal Subramanyam

**Affiliations:** ^1^ Department of Plant Sciences, School of Life Sciences, University of Hyderabad, Hyderabad, Telangana, India; ^2^ Novelegene Technologies Pvt. Ltd, Genomics division, Hyderabad, Telangana, India

**Keywords:** carotenoids, chloroplast, *Dunaliella salina*, photosystems, reactive oxygen species, transcriptome

## Abstract

Understanding the molecular mechanisms of environmental salinity stress tolerance and acclimation strategies by photosynthetic organisms facilitates accelerating the genetic improvement of tolerant economically important crops. In this study, we have chosen the marine algae *Dunaliella (D.) salina*, a high-potential and unique organism that shows superior tolerance against abiotic stresses, especially hypersaline conditions. We have grown the cells in three different salt concentrations 1.5M NaCl (control), 2M NaCl, and 3M NaCl (hypersaline). Fast chlorophyll fluorescence analysis showed increased initial fluorescence (Fo) and decreased photosynthetic efficiency, indicating hampered photosystem II utilization capacity under hypersaline conditions. Also, the reactive oxygen species (ROS) localization studies and quantification revealed elevated accumulation of ROS was observed in the chloroplast in the 3M condition. Pigment analysis shows a deficit in chlorophyll content and increased carotenoid accumulation, especially lutein and zeaxanthin content. This study majorly explored the chloroplast transcripts of the *D. salina* cell as it is the major environmental sensor. Even though most of the photosystem transcripts showed moderate upregulation in hypersaline conditions in the transcriptome study, the western blot analysis showed degradation of the core as well as antenna proteins of both the photosystems. Among the upregulated chloroplast transcripts, chloroplast Tidi, flavodoxin IsiB, and carotenoid biosynthesis-related protein transcripts strongly proposed photosynthetic apparatus remodeling. Also, the transcriptomic study revealed the upregulation of the tetrapyrrole biosynthesis pathway (TPB) and identified the presence of a negative regulator of this pathway, called the s-FLP splicing variant. These observations point towards the accumulation of TPB pathway intermediates PROTO-IX, Mg-PROTO-IX, and P-Chlide, those earlier reported as retrograde signaling molecules. Our comparative transcriptomic approach along with biophysical and biochemical studies in *D. salina* grown under control (1.5 M NaCl) and hypersaline (3M NaCl) conditions, unveil an efficient retrograde signaling mechanism mediated remodeling of photosynthetic apparatus.

## Introduction

*Dunaliella (D.) salina* is a representative of highly salt-tolerant green algae and is well known to show superior tolerance against abiotic stresses. Hence it is an effective model organism to study various acclimatization mechanisms adapted by photosynthetic organisms under high salinity (Katz et al., 2007), which easily get acclimated to a range of salinities from 0.05M to 5M NaCl saturation. The salinity stress affects growth by impaired photosynthetic activity due to the accumulation of harmful oxidative species. Oxidative species are initially generated from the acceptor side of photosystem (PS) I. Hence, there is an emerging understanding of the mechanistic basis for changes in structure in response to environmental cues. *D. salina* is pertinent to our studies because as it grows in high salinity and thus its photosynthetic machinery should evolve to cope with its environment.

Apart from salt tolerance and photosynthetic efficiency, *D. salina* is a promising microalga worldwide for commercially producing an enormous amount of β-carotene. There is a high demand for β-carotene in various industries like pharmaceuticals, food, drugs, etc., The cells under stress turn orange due to the over-accumulation of β-carotene in plastidic oil globules (Ben-Amotz and Avron, 1983). As it has the property to accumulate β-carotene up to 8% of the dry cellular weight, it is a major crop for the production of natural β-carotene in the health and food industry (Borowitzka, 2013). Recently there has been a surge in unraveling the proteome, metabolome, and transcriptome of *D. salina* as it is a halotolerant with high photosynthetic efficiency and a promising crop for β-carotene production. This organism is high potential and unique to produce β-carotene, biofuel, and antioxidants on a commercial scale.

High salt levels impact about 20% of all cultivated land and nearly half of the irrigated land, significantly reducing crop yields to well below their genetic potential (Munns et al., 2006). One realistic strategy to cope with this problem is to increase the salt tolerance of crop plants, either *via* genetic introgression or transgenic technology for gene transfer (Koyama et al., 2001). The effects of salinity stress on crop growth are manifested by impaired photosynthetic capacity. High sodium concentrations in the soil solution impair cell metabolism and photosynthesis by imposing osmotic stress on cell–water relations and increasing sodium toxicity in the cytosol (Brini et al., 2007). Salt stress also impacts other physiological and biochemical processes. A variety of salt tolerance mechanisms have been observed in photosynthesizing organisms. Many plants and cyanobacteria exposed to salt stress produce co-solutes such as sucrose, trehalose, proline, glucosyl-glycerol, and glycine-glutamate-betaine that play essential roles in the salt tolerance of these organisms (Gupta and Huang, 2014). Exogenous proline addition protects plant growth and productivity by reducing free radical production and/or scavenging free radicals. In *Arabidopsis (A.) thaliana*, the Salt-Overly-Sensitive (SOS)1 gene encodes a plasma membrane Na^+^/H^+^ antiporter essential for salt tolerance (Shi et al., 2000). Transgenic *A. thaliana* showed substantial upregulation of SOS1 transcript levels upon NaCl treatment, suggesting post-transcriptional control of SOS1 transcript accumulation (Shi et al., 2003).

Other studies have demonstrated that glyoxalase II overexpression leads to salt tolerance in tobacco and rice plants, and choline oxidase expression plays a role in salt stress tolerance (Singla-Pareek et al., 2008). In the mangrove plant *Bruguiera parviflora*, a 23-kDa PSII-associated protein that plays a major role in oxygen evolution is getting dissociated under high NaCl conditions (Parida et al., 2003). In *Chlamydomonas (C.) reinhardtii*, salt stress-induced changes and acclimation mechanisms have been extensively studied, and morphological studies showed palmelloid formation as a stress-induced behavior (Neelam and Subramanyam, 2013; Devadasu et al., 2023). Also, abscisic acid is a key player in reducing oxidative damage during salt stress (Yoshida et al., 2004). In *C. reinhardtii*, a study on photosynthetic apparatus under salt stress showed oxidative damage to the two PSII core proteins CP43 and CP47 and light-harvesting complex (LHC)II proteins (Ji et al., 2018). A report states that maintenance of efficient photosynthesis by creating fewer and larger PS-I units by *D. salina* under iron deprivation (Varsano et al., 2007). Global transcriptome study in *D. salina* under short-term salinity stress revealed systematic changes in biological processes, including enhancement of photosynthesis, synthesis of glycerol, accelerated protein turnover, carbon fixation, etc (Gao et al., 2021).

Salt stress is a combination of ionic, oxidative, and nutrient deficiency stress, leading to the generation of an enormous amount of reactive oxygen species (ROS), especially in the chloroplast. Chloroplast is one of the major environmental sensors present in cells which helps in acclimation and adaptation under various stress by triggering redox and retrograde signals. Inside the chloroplast, there is an array of ROS-scavenging enzymes and nonenzymatic compounds that work potentially to rebalance the redox homeostasis. But under extreme stress conditions, the balance between the accumulation of ROS molecules and the scavenging system gets disrupted, leading to the damage of biomolecules such as DNA, RNA, and protein. Recent research shows the evolution of a new concept where ROS molecules generated in the chloroplast in a coordinated and controlled manner act as signaling molecules under stress conditions. Once the redox state of the photosynthetic electron transfer (PET) apparatus gets imbalanced, ROS molecules get generated largely in different sites, including the donor and acceptor sides of both PSI and PSII (Foyer and Noctor, 2000; Asada, 2006). The regulation of the redox homeostasis mechanism in chloroplast under salt stress is still unclear. As per previous studies on ROS generation in photosynthetic apparatus, PS I generate hydrogen peroxide (H_2_O_2)_, as well as superoxide (O_2_^-^), and PSII, generates O_2_^-^ alone as signaling molecules. They make the chloroplast an environmental sensor for initiating acclimation mechanisms at transcriptional and post-translational levels (Foyer and Noctor, 2000). Salt stress imposition studies revealed more efficient PSII recovery than PSI. Recent studies on glutathione reductase (GR) knockout plants revealed the redox buffering of chloroplast enabled by a highly reduced glutathione pool to maintain efficient photosynthesis (Müller-Schüssele et al., 2020). The overexpression of glutathione transferase study in tobacco plants proved its enhancement in stress tolerance (Roxas et al., 2000).

Salt stress imposed Chlamydomonas transcriptome study showed impaired photosynthetic activity due to significant downregulation of several PS I proteins (Wang et al., 2018). A previous study on salinity tolerance in Chlorella also showed declined photosystem transcripts such as PsaA, PsaB, PsaC, PsaI, and PsaJ (PS I) and PsbA, PsbB, PsbC, PsbD, PsbE, PsbK, PsbH, PsbM, PsbQ, PsbR and PsbZ (PSII) as well as light-harvesting antenna proteins along with decreased pigment content in the system (Abdellaoui et al., 2019). Understanding metabolome and transcriptome will aid in improving knowledge of salt-induced mechanisms. Without understanding the fundamental mechanism, enhancing its large-scale production is not possible. Environmental pollution, the energy crisis, and the fossil fuel limitation increase the concern for large-scale production of biofuel. Our study aims to improve the fundamental understanding of such potential organism that can be used in several industries effectively.

RNA seq is a powerful and revolutionary tool designed to study the transcriptome of organisms, which helps in understanding the functional aspects and molecular elements at the RNA level. In various organisms, transcriptome study under salt stress unveiled many significant pathways which could have been unheeded (Qiao et al., 2013; Gao et al., 2021; Lv et al., 2021). A transcriptomic study of *D. salina* in Yuncheng Salt Lake, China, revealed several salt-stress responsive genes differentially expressed under different salt conditions, low, moderate, and high NaCl concentrations (Gao et al., 2021). A comparative transcriptomic approach on short-term glycerol and salt-stressed *D. salina* cells revealed unique pathways common to both the stress conditions as well as specific pathways in response to both conditions (Lv et al., 2021). In this study, we have focused on the long-term salt stress responses and acclimation mechanisms in *D. salina* cells by extensive analysis of differentially expressed genes (DEGs) fetched out from the high throughput RNA seq data. Our study explored the chloroplast transcripts and correlation of physiological studies of the *D. salina* cells as it is the primary environmental sensor. Gene expression profile studies revealed the cross-talk between redox and retrograde signaling in the chloroplast of *D. salina* and its significance in long-term acclimation under hypersalinity.

## Materials and methods

### *Dunaliella salina* growth and culture conditions

*D. salina* has been grown photoheterotrophically in different salt concentrations ranging from 1.5M (optimum concentration) and 3M (hypersaline concentration) saturation. Cells were grown in BG11 medium supplied with different NaCl concentrations at 25°C with a photon flux density of 50-60 µmol photons m^−2^ s^−1^ in a 1000 mL bottle shaken at 120 rpm in an orbital shaker. Optical density was measured at 730nm. The cell count was measured using a hemocytometer, followed by chlorophyll estimation based on standard protocol. Cells were harvested from 200 ml of culture in the mid-logarithmic growth phase after 72 h by centrifuging at 4000 rpm without damaging the cells. Immediately after harvesting, the cells have been frozen using liquid nitrogen and taken for RNA isolation. The chlorophyll and carotenoid pigment content in control and treated samples were measured as described earlier (Lichtenthaler, 1987; Porra et al., 1989).

### Fast Chlorophyll *a* fluorescence measurement

Fast chlorophyll (Chl) measurements were measured using a Handy PEA fluorimeter to check the maximum quantum efficiency of PS II with incident light of high intensity (3000 µmol photons m^−2^ s^−1^) in a very short period of 1 sec. The photosynthetic efficiency has been recorded as the fluorescence parameter Fv/Fm, whereas Fm is the maximum fluorescence and Fv is the variable fluorescence calculated by subtracting initial fluorescence Fo from Fm. The Chl fluorescence kinetics is represented as an induction curve in which peaks are denoted by four letters O, J, I, and P. O represents the initial fluorescence (Fo); O–J phase that reflects the reduction of the primary quinone acceptor of PSII, Q_A_ to Q_A_^−^; J–I and I–P phases involved in the reduction of PQ pool as well as electron acceptor side of PSI; at maximum fluorescence level (Fm), reduced electron carriers in between the PSII reaction center and NADP (Kodru et al., 2015).

### Confocal imaging of *D. salina* cells

*D. salina* grown under different salt conditions has been harvested after 3 days of growth for confocal imaging. A Leica super-resolution microscope has been used for imaging the cells to see morphological changes. Cells were immobilized using 0.025% iodine solution and observed in 100x magnification for live cell imaging and autofluorescence and images captured in 5nm scale in all conditions.

### ROS localization and imaging

*D. salina* cells were grown under 1.5M, 2M, and 3M conditions and were harvested on the third day and pelleted by centrifugation at 3000 rpm for 10 min. H_2_DCFDA dye is used to stain ROS, and 500 µM stock solution of dye was prepared in DMSO and prepared a dilution of 10µM in water. The cells were treated with 5µM dye and incubated in the dark for 1 h. Afterwards washed three times using its growth medium. H_2_DCFDA fluorescence was detected at 500-530nm wavelength and an optical filter of 600nm. Samples were viewed with a magnification of 100x oil immersion. Also, the treated cells were used for fluorescence measurement using a plate reader, an excitation wavelength of 485 nm and an emission wavelength of 530nm.

### Isolation of thylakoids

The cells were harvested after 72 h of growth by centrifugation at 4500 rpm for 5 min and washed once with buffer containing 30mM Tricine-NaOH (PH-8), 15mM NaCl, and 0.4 M sucrose. Discarded the supernatant after washing and resuspended the pellet within the above buffer. Sonicate the resuspended cells with an amplitude of 25% for 4 cycles of 10 sec on and 50 sec off. Centrifuge the suspension at 12000 rpm for 10 min and then take the supernatant for ultracentrifugation at 45000 rpm for 2h. after centrifugation, resuspends the pellet in buffer containing 30mM Tricine-NaOH (PH-8), 100mM NaCl, and 0.4 M sucrose buffer and incubate it for 30 min, then repeat the ultracentrifugation as previously. Collect the pellet and store it in the 30mM Tricine-NaOH (PH-8), 15mM NaCl, and 0.4 M sucrose buffer (Perez-Boerema et al., 2020).

### Western blot analysis

The isolated thylakoid sample from control, 2M and 3 M grown cells are used to separate the photosystem proteins using SDS-PAGE (Schägger and Von Jagow, 1987). A 12% resolving gel is used for separating the denatured protein samples. Then the polypeptides separated in the gel are taken for semi-dry immunoblot transfer apparatus (Biorad), and the polypeptides are transferred to a nitrocellulose membrane. The membrane was kept for incubation primarily overnight, and the secondary antibody (antibody anti-rabbit HRP - conjugated antibody) was for 1h. The primary antibodies used in dilutions PSII: PsbA (1:10,000) PsbD (1:5000), PsbC (1: 3000), PsbB (1: 10000) PSI: PsaA (1:5000), PsaB (1: 1000) PsaD (1:10000) PSI LHCs: LhcA1 (1:5000), LhcA2 (1:5000) AtpC (1: 10000). The images were detected using ChemiDoc, Bio-Rad.

### HPLC analysis of pigment composition

Pigments from the harvested *D. salina* cells grown under control and treated conditions have been identified using high-performance liquid chromatography (HPLC) as described (Croce et al., 2000), with some modifications. First, pigments were extracted using 100% acetone and centrifuged at 10000 rpm for 10 min to remove unwanted materials. The supernatant was thermal vacuum dried for 2 h. The dried pigments were solubilized with 400 µl of mobile phase solvent composed of acetonitrile: acetone: methanol (70: 20:10) and filtered using 0.4-micron filters. The analysis was carried out using HPLC (Shimadzu) system on the C18 column. Chl a and Chl b peaks have been extracted at 662 and 642 nm, respectively. Also, different carotenoid pigments, β-carotene, zeaxanthin, lutein, xanthophyll, and violaxanthin, were obtained at 453nm, 481nm, 446nm, 443nm, and 444nm, respectively.

### RNA seq analysis

The frozen sample from three independent biological replicates was immediately taken for grinding using a motor and pestle without allowing the sample to thaw. The disrupted cells were isolated for RNA using an RNeasy plant mini kit (Qiagen). The isolated RNA sample has been taken for quantity and quality check using an Agilent 2100 Bioanalyzer (Agilent Technologies, Palo Alto, CA, United States) and agarose gel electrophoresis to observe if any RNA degradation occurs. After quality and quantity checks of the RNA from three replicates, each from control and treated samples, have been submitted to Novelegen company for Illumina NovaSeq 6000 sequencing. The raw read sequences obtained from the sequencer have been processed using Fastq for quality control check. Approximately 75 million base pairs read from each sample have passed the Q20 and Q30 quality control checks with above 90% and 53% GC content; these reads have been separated as high-quality for further processing. All the high-quality reads were mapped against the reference generated by Trinity using the Trinity pipeline (bowtie2) genome assembler. After mapping the reads, the BAM file containing the mapping information used for quantifying reads was generated. The downstream analysis requires the number of reads mapping to each genomic feature, for example, each exon or each gene. Read summarization (Quantification) has been conducted using the RSEM program, and gene expression level (FPKM) was generated using this. Among each sample, approximately 40 million reads were mapped, and 25 million reads were aligned. Based on the global gene expression level principal component analysis (PCA analysis) and correlation analysis have been conducted (**Supplementary Figures 1A, B**). DEGs were generated among the mapped genes using the Dseq2 program with a fold change of ≥ 0.5 and FDR ≤ 0.05 (**Supplementary Figure 2A, B**). The transcriptomic data was uploaded to NCBI under the Bio project ID PRJNA946036 and submission ID SUB12963156.

### qRT PCR analysis

The accuracy of differentially expressed genes has been validated using qRT-PCR analysis (**Supplementary Figure 3**). Among the most upregulated genes, 10 were randomly selected and primers were designed (**Supplementary Table 1**) using primer 3, and primer parameters were rechecked using oligocalc. cDNA synthesis has been done using Primescript™ 1^st^ strand synthesis kit. Ribosomal protein L2 served (RPL2) as the reference gene for qPCR analysis.

## Results and discussion

### Growth and photosynthetic efficiency of *D. salina*

**Figure 1:**
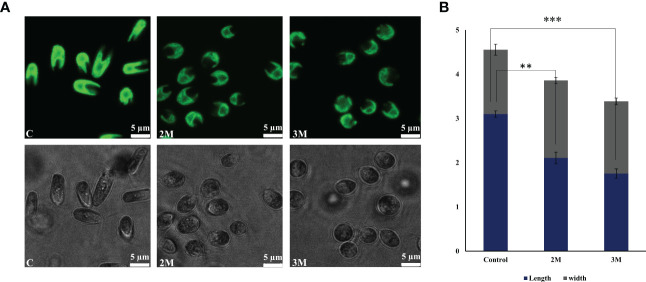
Morphological changes observed in *Dunaliella salina*. **(A)** Confocal images of cells were observed under control, 2M, and 3M hypersaline conditions. **(B)** Graph representing cell length and width change under control, 2M, and 3M hypersaline conditions. The means ± SD were calculated (NaCl) for three biological replicates. Statistical comparison was performed using one- way analysis of variance (ANOVA) followed by the Tukey multiple comparison tests, and p-values obtained are indicated asterisks (∗∗∗p < 0.001, ∗∗p <0.01).

*D. salina* cells were grown in various salt concentrations (1.5 to 3 M NaCl) to understand the remodeling of photosynthetic apparatus as a salt tolerance mechanism to operate photosynthesis. Here the 1.5M growth is considered a control, and this concentration is an optimal growth condition. In the 2M growth condition, even though cells are growing slowly, they are acclimatizing within 3 days, and in 3M, the growth is severely impaired (**Supplementary Figures 4A–D**). The culture color turns from light green to yellowish green from 1.5M to 3M, respectively (**Supplementary Figure 4C**). Pigment analysis showed a moderate decrease in chlorophyll pigments and an increase in the carotenoid pigments under hypersaline (**Supplementary Figure 5**); the same trend has also been observed in the isolated thylakoid membranes. Enhanced carotenoid pigments must be an efficient strategy in the acclimation process involving excess energy quenching and non-enzymatic ROS scavenging. The cell growth in the control and 2M treated conditions reached the stationary phase after 120 h of growth and 3M condition showed an actively growing phase of 164 h. When the cells reach the stationary phase, the control culture contains 1.73x10^6^ cells. The 2M and 3M have 1.38 x10^6^ and 0.97 x10^6^ cells, respectively. The morphology has been observed to be a round shape with reduced size in hypersaline (**Figures 1A, B**).

**Figure 2:**
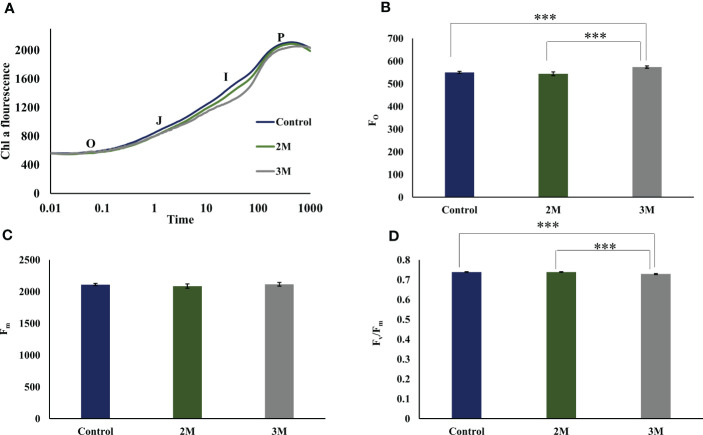
Fast chlorophyll a fluorescence analysis of control, 2M and 3M NaCl. **(A)** OJIP transient data was obtained from the three conditions. O represents the initial fluorescence O, initial fluorescence (Fo); OJ phase that reflects the reduction of the primary quinone acceptor of PSII, Q_A_ to Q_A_^−^; JI and IP phases involved in the reduction of PQ pool as well as electron acceptor side of PSI; at maximum fluorescence level (Fm). **(B)** Initial fluorescence (Fo) from control, 2M, and 3M conditions. **(C)** Maximum fluorescence (Fm)from control, 2M, and 3M conditions. **(D)** Maximum photosynthetic efficiency (Fv/ Fm) in control, 2M and 3M conditions. The means ±SD were calculated for 3 biological replicates. Statistical comparison was performed using one- way analysis of variance (ANOVA) followed by the Tukey multiple comparison tests, and p-values obtained are indicated asterisks (∗∗∗p < 0.001)

The chlorophyll fluorescence induction curve is an efficient and fast method to analyze the photosynthetic efficiency of oxygen-evolving photosynthetic organisms. The Chl fluorescence induction kinetics shows the photochemical activity of PSII in a time course of 1 sec and gives OJIP transient data (**Figure 2A**). ‘O’ represents the initial fluorescence (F_O_), and P represents the maximum fluorescence (F_m_) when it reaches its maximum. J step represents the formation of Q_A_^-^Q_B,_ and I step reflects the formation of Q_A_^-^Q_B_^-^ form. Finally, the P reflects the accumulation of Q_A_^-^Q_B_^2-^ form (Strasser, 1992). Chl a fluorescence data has shown almost similar photosynthetic efficiency after three days of growth in optimum condition as well as in the hypersaline condition of 3M. Comparing the transient fluorescence recorded in hypersaline grown cells to optimum grown cells shows a slight increase in the F_O_ (**Figure 2B**) and a marginal decrease in the PS II efficiency (Fv)/Fm) (**Figure 2D**) without change in F_m,_(**Figure 2C**). Perhaps, this could be because of the decrease in the overall utilization of trapped energy for photochemistry. Also, the slight dip observed in the I phase reflects the reduced rate of reoxidation of the PQ pool.

### Transcriptomic analysis shows efficient acclimation strategies at the chloroplastic level

**Figure 3:**
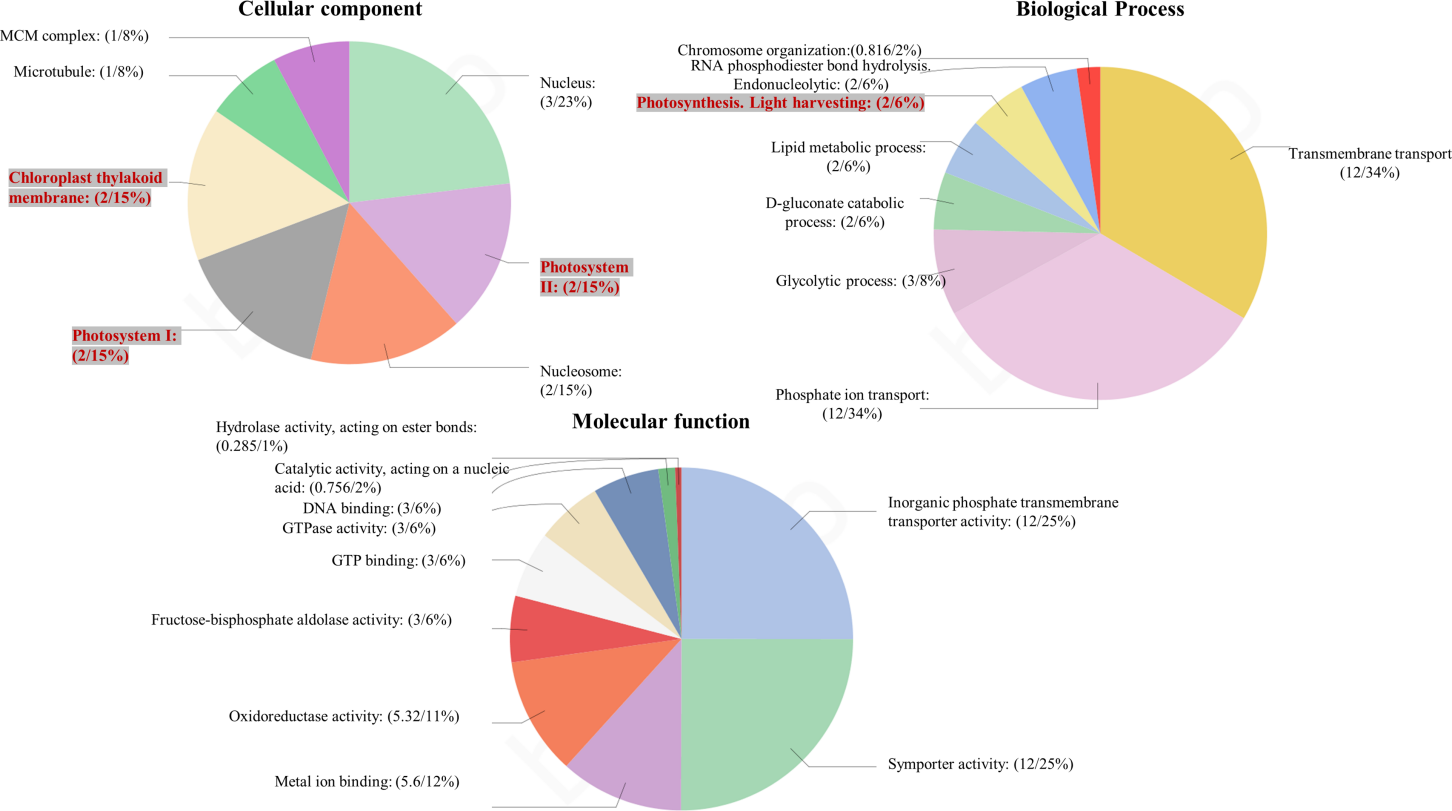
Gene ontology analysis of differentially expressed genes using BLAST2 GO software. The percentage of total upregulated transcripts in different GO categories biological processes, molecular function, and cellular components.

**Figure 4:**
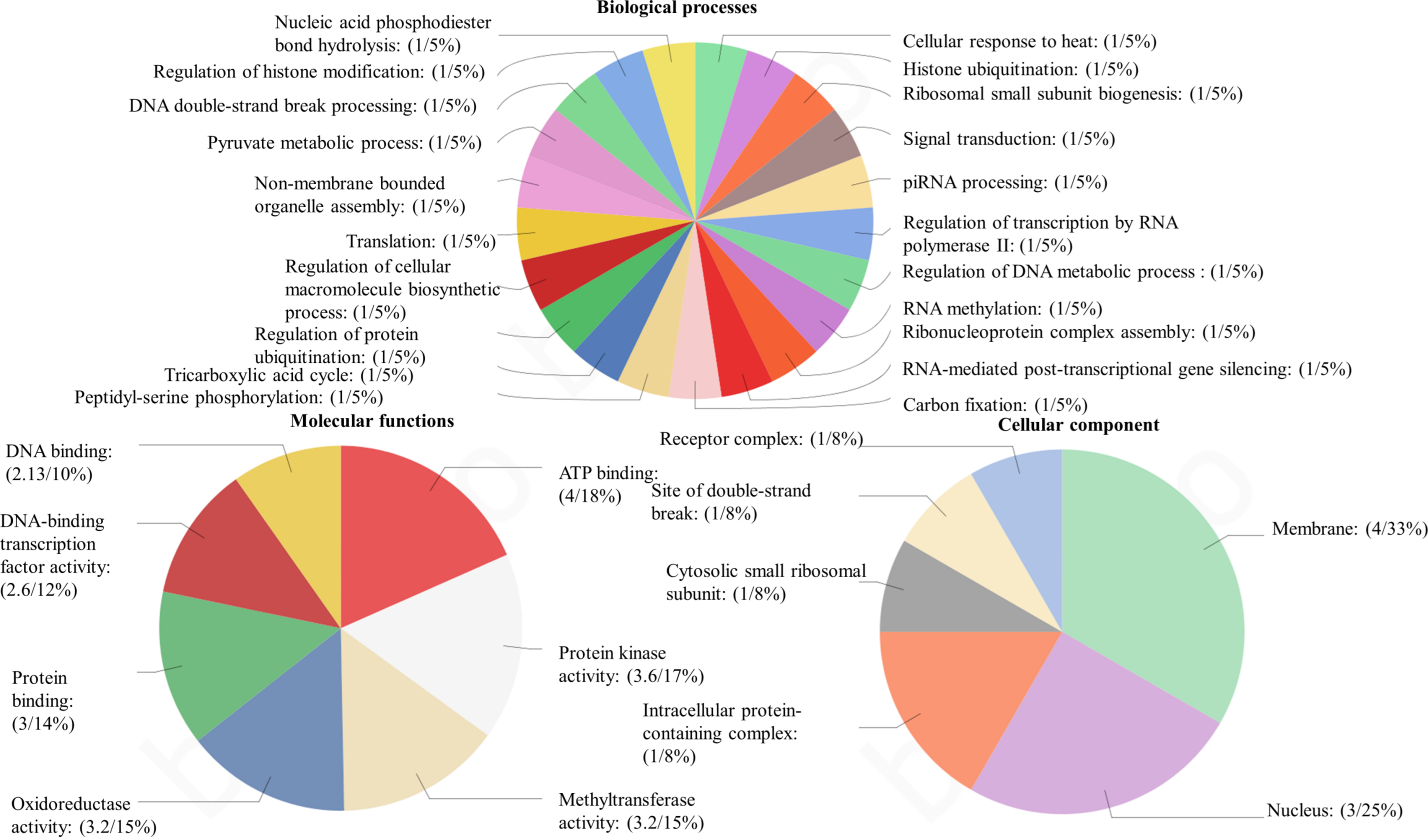
Gene ontology analysis of differentially expressed genes using BLAST2 GO software. The percentage of total downregulated transcripts in different GO categories biological processes, molecular function, and cellular components.

**Figure 5:**
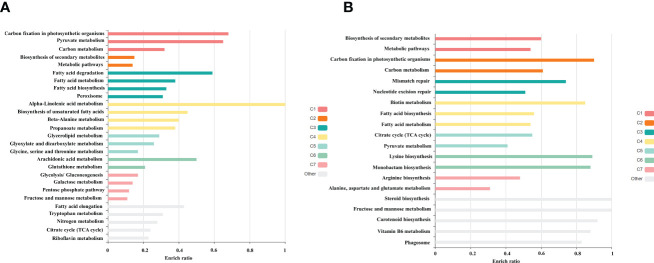
Gene enrichment analysis of differentially expressed gene using KOBAS 3.0 software **(A)** Upregulated **(B)** Downregulated transcripts.

Among the obtained transcripts significant DEGs were separated, setting a fold change above 1.5. GO enrichment analysis of all the DEGs was performed using Blast2GO software. Both the upregulated (**Figure 3**) and downregulated (**Figure 4**) transcripts were categorized into three GO functional groups, cellular component, molecular function, and biological process. More than 50% of the transcript categorized under cellular component was localized in the chloroplast, especially components of photosystem and proteins located in the thylakoid membrane. The other major part of the transcriptome showed a significant uptrend localized in the nucleus, minichromosome maintainance complex (MCM), and nucleus indicating cellular reorganization. Transmembrane transport shows a significant role under hypersaline conditions, especially the phosphate ion transporter and symporter activity. Pathway enrichment analysis of both upregulated (**Figure 5A**) and downregulated (**Figure 5B**) transcripts have been done using KOBAS software to select specific pathways showing significant changes. Among the pathways fetched out, carbon fixation, secondary metabolite synthesis, carotenoid biosynthesis, and fatty acid metabolism show substantial changes. Further, gene ontology (GO) analysis and pathway enrichment primarily indicate an efficient chloroplast signaling mechanism in response to the hypersaline condition as a significant environmental sensor. Especially photosystem subunit level changes showed in GO analysis must play a key role in salinity acclimation. Along with that, carotenoid biosynthesis and elevated secondary metabolite synthesis help in acclimation and repair processes.

### Photosystem I remodeling and role of chloroplast Tidi

PS I function takes a short span of time for charge separation and conversion of excitation energy into radical pair (Wilhelm and Wilhelm., 1993). PSI is a multi-pigment-protein complex span over thylakoids with extrinsic, intrinsic, and transmembrane subunits, which together function as a strong redox system to convert NADP^+^ to NADPH. The antenna molecules absorb photons and get excited, leading to charge separation between P700 and A_0_ molecules. The excited electron subsequently reduces a chain of oxidized molecules A1, FeX, FeA, and FeB iron-sulfur clusters, and finally, it reduces the ferredoxin molecule. Ferredoxin transfer electron to NADP+ to generate NADPH (Golbeck, 1992). PSI is considered the most stable redox catalyst because of its shorter span of charge separation due to its tightly packed pigment molecules and symmetrical arrangement of Ao and RC Chl molecules. Being a fast redox system, PSI is less prone to photoinhibition under stress conditions when compared to PSII (Caffarri et al., 2014). Our transcript study also points towards PSI’s potent and efficient remodeling to rebalance the excitation energy distribution in the photosynthetic apparatus under hypersaline conditions.

**Figure 6:**
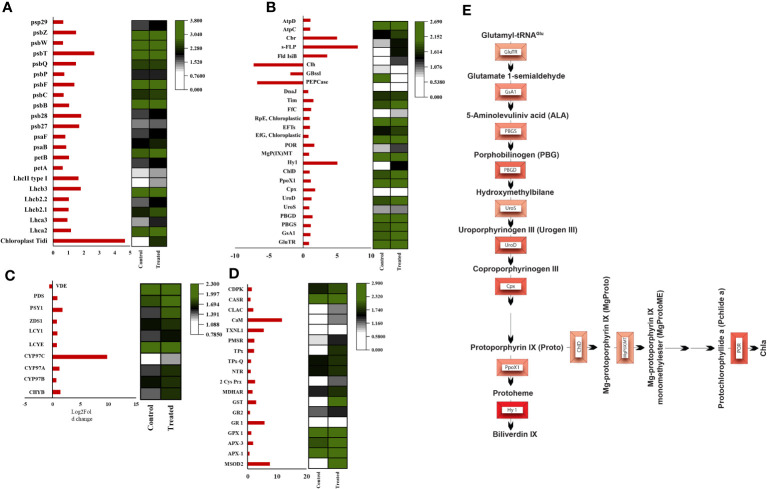
Heatmaps of significant differentially expressed genes. **(A)** Heatmap of photosystem-associated transcripts, **(B)** Heatmap of tetrapyrrole biosynthesis pathway-associated transcripts **(C)** Heatmap of carotenoid biosynthesis associated transcripts, **(D)** ROS and calcium signaling associated transcripts, **(E)** Pathway representation of all the upregulated (intensity of red representing the foldchange) transcripts involved in the tetrapyrrole biosynthesis pathway.

Analysis of DEGs reveals that the transcript level of PS I-related proteins has been showing a general trend of moderate upregulation of all proteins and a thylakoid transcript chloroplast Tidi was showing a significant increase (**Figures 6A, B** and **Supplementary Table 2A**). The transcriptional regulation of photosystem-related proteins varies in different photosynthetic organisms due to ecological and evolutionary reasons. In the model organism, *C. reinhardtii*, the transcriptional level of PS I light-harvesting proteins was reported as downregulated under salt stress conditions (Wang et al., 2018). Further, in Ulva, the subunits of PS I and II as well as all the LHC transcripts found to be significantly downregulated in salt stress.

Among the photosynthetic transcripts analyzed in both PSI and PSII from DEGs, the most upregulated transcript observed was chloroplast Tidi (TRINITY_DN12221_c0_g1_i9), showing a log2 fold change of 4.6. Also, most of the PSI light-harvesting proteins showed a moderate up-regulation. Chloroplast Tidi is a 45kD chlorophyll-binding protein localized in the thylakoid membrane reported earlier, specifically iron deficit inducible in *D. salina* (Varsano et al., 2007). The recent transcriptome analysis of short-term hypersaline-treated *D. salina* reported downregulation of the LHC proteins, and there was no report of chloroplast Tidi induction in shock treatment (Lv et al., 2021). When comparing this scenario with our study, chloroplast Tidi upregulation must play a key role in the long-term hypersaline acclimation of photosynthetic apparatus in *D. salina* and moderate upregulation of all other subunits. Recently the *D. salina* PSI structure has been elucidated as a minimal PSI structure with 11 subunits, including PsaA-PsaF, PsaI, and the LHC proteins Lhca1- Lhca4, without PsaG, PsaH, PsaI, PsaK, PsaL, and PsaO (Perez-Boerema et al., 2020; Caspy et al., 2023). From the above studies, the missing subunits of PSI structure can associate with additional Lhc proteins under certain physiological conditions. They suspect its functional implication as it could be for reducing the association in particular conditions or promoting specific remodeling and distinct interactions under certain conditions. In line with this hypothesis, chloroplast Tidi is associated with the PSI-LHCI complex with its unique N terminal domain under iron deficit conditions. Its accumulation is in correlation with decreased PSI subunits. Low-temperature fluorescence studies and BN-PAGE analysis of photosystem supercomplexes from iron deficit cells revealed an increase in PSI supercomplex size and Chl a fluorescence. They suspect that this is contributed by the CAB-like protein chloroplast Tidi by its association with PSI to rebalance the energy distribution between PSII and PSI in stress conditions (Varsano et al., 2006).

**Figure 7:**
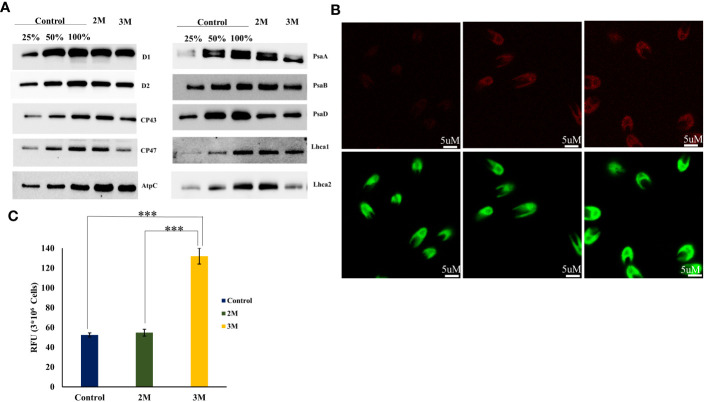
ROS accumulation in chloroplast and oxidative degradation of photosystem-associated proteins. **(A)** Western blot analysis of photosystem-associated proteins isolated from the thylakoid sample of control and hypersaline condition. **(B)** Confocal localization of total ROS accumulated in control, 2M, and 3M NaCl using Leica high-resolution microscopy technique, the cells incubated in H_2_DCFDA for 1 h. **(C)** Total ROS measured using UV-visible spectrophotometry. Statistical comparison was performed using one- way analysis of variance (ANOVA) followed by the Tukey multiple comparison tests, and p-values obtained are indicated asterisks (∗∗∗p < 0.001).

Our immunoblot studies of PS I subunits showed that PsaA undergoes more degradation than PsaB, and the PsaF subunit was unaffected. Among LHCs, Lhca2 and Lhca1 showed decreased expression levels, probably due to oxidative damage (**Figure 7A**). Also, the ROS localization study and elevated level of total ROS was observed in the hypersaline conditions compared to the control condition (**Figures 7B, C**). These elevated ROS levels must lead to the degradation of photosystem subunits. But both the western blot and transcript level analysis of ATP synthase subunits was showing an upregulated trend in hypersaline conditions. In hypersaline conditions, *D. salina* requires a high amount of energy to execute many acclimations mechanisms such as a high amount of glycerol production, cellular rearrangement, pigment production, etc., for its survival. So uninterrupted ATP synthase activity is essential to meet the energy requirements of the organism. The system must be protecting ATP synthase from degradation for better photosynthetic performance by uninterrupted electron transport through the photosynthetic apparatus. Compared with an earlier report of enhancement in PSI level in short-term salt treatment, our study shows the degradation of a few PSI subunits in the long term, which could be promoting the chloroplast Tidi association and enlargement in PSI particle size similar to iron deficit condition. The change in antenna complexes, the remodeling of the PSI occurred due to acclimation by over-expression of TiDi. Also, rebalancing the redox state of PSI and PSII as a long-term acclimation of the photosynthetic apparatus of *D. salina* under hypersaline conditions. PSI structure of *D. salina* is suspected to be evolved in a way to adapt to extreme saline conditions as well as other abiotic stress as it has an oceanic origin; structural plasticity of PSI structure must be playing a pivotal role in such acclimation processes under various physiological conditions specifically in *D. salina.*

### Photosystem II assembly and repair regulation

Analysis of photosystem transcripts’ expression level proclaims a general trend of upregulation among reviewed DEGs (**Figure 6A, B** and **Supplementary Table 2A**). PS II is a large transmembrane protein complex composed of several protein subunits and pigment molecules localized in the thylakoid membrane’s stromal and luminal sides. According to the recent structure of PSII of C2S2M2 of *C. reinhardtii* comprises 3 LHCII trimers, 2 major antennae CP26, and CP29 surrounding a dimer core and peripheral protein subunits (Shen et al., 2019). Algal PSII is elucidated in cryo-EM structures as having extra light-harvesting antenna molecules in the photosystem. This could be due to its oceanic origin and underwater ecological niche. Photosystems undergo various types of repairs and remodeling under unfavorable conditions to acclimate and adapt photosynthetic apparatus. The protein-pigment complexes undergo degradation, repair, and remodeling depending on the type and intensity of stress. Photooxidative damage of PSII is an unavoidable evil that also helps in protecting the other part of the photosynthetic apparatus from further damage. PSII is more prone to photooxidative damage because of its monodirectional electron transfer, weaker pigment molecule interactions, and distorted arrangement of the porphyrin plane of all molecules bound to the PSII reaction center (Wilhelm and Wilhelm., 1993).

Several PSII-associated proteins showed transcriptional upregulation in our data, especially PSII repair and assembly proteins. One interesting transcript showing a significant upregulation with a log2 fold change of 2.6 was carotenoid biosynthesis-related protein (CBR) (TRINITY_DN2118_c0_g1_i13). CBR is an algal homolog of the ELIP family protein in plants, which was reported first in *D. bardawil* as high light-induced protein functions in photoprotection (Adamska et al., 1992). It is reported that CBR accumulation is in parallel with carotenogenesis. The earlier report proposed a physical association between the pigments and CBR protein to protect disassembled PSII under light stress (Levy et al., 1993). Our HPLC data and carotenogenesis-related transcripts data align with the upregulation of CBR transcript. Also, the disassembly and degradation of PSII subunits D1, D2, CP43, and CP47 proteins in 3M salt conditions were observed through western blot analysis (**Figure 7A**). This is probably due to oxidative degradation by the excess accumulated ROS in the chloroplast. It is tempting to suggest that CBR accumulation and upregulated carotenoid synthesis point towards the protective role of CBR in hypersaline-grown *D. salina* photosynthetic apparatus.

Along with this, the carotenoid hydroxylating enzymes, which play a role in the generation of xanthophyll molecules from α and β carotenoids, show marked upregulation in 3 M salt. Also, the transcript data support the protective function of CBR binding with xanthophyll molecules in long-term hypersaline conditions. Another transcript showing a significant upregulation was flavodoxin IsiB, which consists of electron shuttles in the acceptor side of PSI complex functions similar to ferredoxin molecules in prokaryotes and some oceanic algae (Lodeyro et al., 2012). These molecules contain FMN prosthetic groups instead of Fe-sulfur clusters to participate in oxidation-reduction reactions in photosynthetic apparatus. The presence of flavodoxin molecule is an adaptive trait with the remodeling in photosynthetic apparatus to acclimate under iron deficit as well as some other abiotic stress (Lodeyro et al., 2012). In *D. salina* these molecules must be playing a pivotal role in remodeling and balancing the redox reactions replacing the ferredoxin molecules in hypersaline conditions.

Further analysis of PSII-associated protein transcripts revealed upregulation of several repair and assembly factors such as Psb27 (TRINITY_DN2037_c0_g1_i6), Psb28 (TRINITY_DN2123_c0_g1_i8), PsbZ (TRINITY_DN7535_c0_g1_i1), etc., Basically Psb28 and Psb27 are two major PSII biogenesis factors function in different steps in protecting the assembly intermediates which are more prone to damage in stress conditions (Dobáková et al., 2009; Boehm et al., 2012). This gives insight into the efficient biogenesis of the PSII complex and the protection of the intermediate complex to acclimate under hypersaline-triggered photooxidative damage of photosynthetic apparatus. Psb28 plays a major role in the association of CP47 with the reaction center intermediate forming the primary PSII assembly intermediate RCP47 complex. Psb27 protects free unbound CP43 from damage before incorporating it into the CP47 complex. It also suggested a possible function of Psb27 in restraining the flexibility of the CP43 E loop, which has an indispensable role in OEC assembly, for conformational changes for further OEC assembly (Komenda et al., 2012). PsbZ was also showing a moderate upregulation in our system, and a key role of Psbz as a small peripheral subunit that connects the core is proposed. The other subunits play a role in NPQ by inducing elicitation mechanisms in the core to dissipate excess excitation energy. This mechanism has not yet been well studied in terms of Psbz’s contribution to the NPQ mechanism. This must be vital in stabilizing hypersaline-triggered damages in the *D. salina* photosynthetic apparatus. PsbT and PsbM are two important subunits engaged in assembly and protection, which showed moderate upregulation in high saline conditions. PsbM functions in the assembly of CP47 monomer complexes during the biogenesis of PSII. PsbM does not bind with this intermediate without PsbT (Iwai et al., 2004). Mutant studies in a double mutant of PsbM and PsbT showed increased photodamage and decreased recovery rate of photosynthetic apparatus, suggesting their inevitable role in the assembly and repair process (Bentley et al., 2008). Therefore, a moderate increase in PsbM and PsbT subunits involved in photoprotection against hypersaline in *D. salina*.

Oxygen evolving complex is an irreplaceable component of PSII which take part in the photolysis of water. In our study, both OEE2 (PsbQ) (TRINITY_DN19192_c0_g3_i1) and OEE3 (PsbP) (TRINITY_DN5262_c0_g1_i21) showed moderate upregulation. These are the two extrinsic subunits of the oxygen-evolving complex, which are involved in the binding of the cofactor’s chloride (Cl^-^) and calcium (Ca^-^) (Mayfield et al., 1989). In other halophytic plants such as *Bruguiera gymnorrhiza* and *Salicornia europaea* (Momonoki et al., 2009), it has been reported that instead of OEE2 and OEE3, OEE1 was showing significant upregulation. So, among halophytes, the strategies differ in acclimation to protect and functionalize the photosynthetic apparatus. Apart from core subunits and assembly factors, light-harvesting complex proteins were also showing a general trend of upregulation, Lhcb3 (TRINITY_DN1444_c0_g1_i22), LhcII type I (TRINITY_DN1444_c0_g1_i14), Lhcb2.1 (TRINITY_DN1444_c0_g1_i1), Lhcb 2.2 (TRINITY_DN3207_c1_g1_i1) all lhcs and core. This indicates that long-term growth in the high salt of *D. salina* has different kinds of acclimation than freshwater algae and land plants. High salt is often has a deleterious effect on freshwater algae or plants, especially when the water oxidation complex (WOC) is damaged drastically (Gupta, 2020).

### Carotenoid biogenesis

*D. salina* is known for its escalated accumulation of β-carotene under different abiotic stress conditions such as high light, UV exposure, nitrogen starvation, glycerol stress, and salinity stress. Carotenoids are the indispensable components of photosynthetic apparatus from bacterial photosystems to plant photosystems. Carotenoids are an integral component of light-harvesting complexes in the multi-protein photosystem complex. They play a crucial role in light harvesting and photoprotection by quenching the triplet chlorophyll, dissipating excess excitation energy in the system, and scavenging ROS. It has elucidated that specific xanthophyll molecules have a peculiar and precise function in photoprotection. α-carotene branch-derived lutein, β- carotene branch-derived zeaxanthin, and antheraxanthin have an irreplaceable role in dissipating excess excitation energy to avoid photodamage (Niyogi et al., 1997).

**Figure 8:**
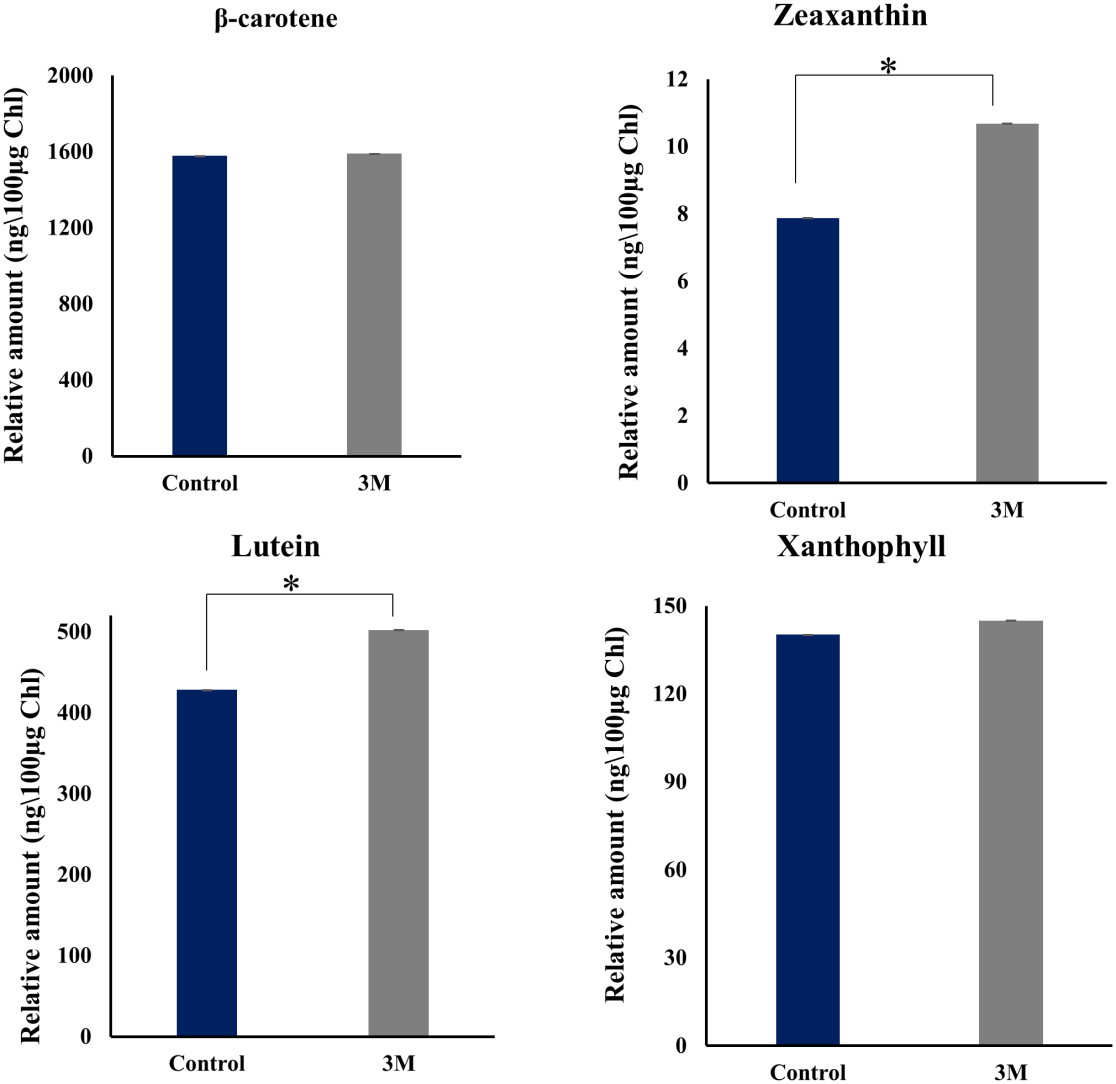
HPLC analysis of change in pigment composition from the thylakoid sample isolated from control, 2M, and 3M NaCl. Different carotenoid pigments are β-carotene, zeaxanthin, lutein, xanthophyll, and violaxanthin at 453nm, 481nm, 446nm, 443nm, and 444nm, respectively. The means ± SD were calculated for 3 biological replicates. Statistical comparison was performed using one- way analysis of variance (ANOVA) followed by the Tukey multiple comparison tests, and p-values obtained are indicated asterisks (*p < 0.001).

Interestingly, analysis of transcripts from carotenoid biogenesis revealed upregulation of most of the enzymes involved in α- carotene and β-carotene branch-derived pigments synthesis (**Figure 6C** and **Supplementary Table 2C**), which aligns with the upregulation of the chloroplastic Cbr gene. Both these point towards an efficient acclimation of photosynthetic apparatus. Cytochrome P-450 hydroxylase (CYP97C1) (TRINITY_DN4283_c0_g1_i7) was observed as the most abundant transcript, and it has shown a fold change of 9.7. This enzyme has carotenoid β- ring hydroxylating activity responsible for lutein accumulation (Inoue, 2004). Along with CYP97C1, two more hydroxylating enzymes show a moderate upregulation, CYP97A, and CYP97B. These three enzymes are well studied in *D. bardawil* and have been reported for their hydroxylating activity and conversion of α-carotene and β-carotene into lutein (Liang et al., 2020). CYP97C can hydroxylate β- carotene and α-carotene; however, CYP97A can hydroxylate only the β-carotene ring. In fact, CYP97B has only minor activity in the hydroxylation of α- carotene. Also, our HPLC data from the thylakoids from *D. salina* grown under 3M NaCl condition shows increased lutein production (**Figure 8**). These results reveal an essential role of lutein in long-term acclimation under hypersalinity conditions. The enzyme considered to be the rate-limiting enzyme in carotenoid biogenesis, phytoene synthase (TRINITY_DN43_c1_g2_i1) was also observed to be moderately upregulated in long-term treatment. This might be the reason for the system’s upregulation of total carotenoid content. β-carotene 3-hydroxylase (BCH) (TRINITY_DN10949_c0_g1_i2), which converts β-carotene to zeaxanthin by hydroxylation of its 3^rd^ position in both the rings (Cazzonelli and Pogson, 2010) and this will be the precursor of many other important xanthophylls plays a crucial role in nonradiative energy dissipation under certain conditions showed a moderate upregulation. Also, in *D. bardawil* BCH has explained the hydroxylation of β-rings of α- carotene as well as β- carotene and leads to the accumulation of lutein. Considering the upregulated activity of BCH, CYP97A, CYP97B, CYP97C, and increased accumulation of lutein in HPLC analysis gives an insight into the presence of multiple pathways involved in lutein accumulation. It is involved in nonradiative energy dissipation under long-term hypersaline acclimation of the *D. salina* photosynthetic apparatus.

### Cross-talk between ROS and calcium signaling mechanism

The proper functioning of chloroplast needs coordinated action of both the nucleus and plastid genome signaling. When the redox poise in the chloroplast is harnessed, it sends messages to the nucleus to initiate signaling mechanisms that rebalance the system, called retrograde signaling (Nott et al., 2006). The regulative mechanisms executed by retrograde signaling can be divided into two categories biogenic control (chloroplast biogenesis associated) and operational control (triggered by environmental stimuli) (Pogson et al., 2008). Retrograde signaling has been well explored in Arabidopsis by isolating and characterizing genome uncoupled (GUN) mutants, which are still nascent in the algal system. Analyzing the *D. salina* transcriptome under hypersalinity tempts to propose strong and efficient retrograde signaling to reestablish the chloroplast’s redox state. The uptrend pattern shown in the expression level of ROS scavenging and calcium signaling transcripts promulgates the idea of cross-talk between both the signaling mechanisms and the establishment of chloroplast retrograde signaling.

Analyzing the transcriptome, non-enzymatic, and enzymatic ROS scavenging system shows a significant uptrend pattern (**Figure 6D** and **Supplementary Table 2B**). Many studies unveiled the importance of different antioxidant enzymes such as catalase (CAT), superoxide dismutase (SOD), ascorbate peroxidase (APD), and glutathione reductase (GR) in salt tolerance (Cejudo et al., 2021). Among those enzymes, manganese superoxide dismutase, which acts as the major ROS scavenging enzyme in the mitochondria (TRINITY_DN4937_c1_g1_i8), showed a maximum upregulation of 7-fold. Our analysis detected different isoforms of ascorbate peroxidase, and APX3, a peroxisomal enzyme, showed (TRINITY_DN3502_c0_g1_i122) an increase of 1.8 log2 fold change. From previous mutant studies in Arabidopsis, APX3 was proposed to be insignificant in oxidative stress (Narendra et al., 2006). However, our transcriptome data propose an important role of APX3 under oxidative stress. Enzymes involved in the glutathione metabolism, including Glutathione S transferase (TRINITY_DN11514_c0_g1_i24), glutathione peroxidase (TRINITY_DN16267_c0_g1_i9), glutathione reductase (TRINITY_DN6033_c0_g2_i13), and monodehydroascorbate reductase (TRINITY_DN131_c0_g1_i6) are significantly upregulated which reveals an efficient ROS detoxification system switch by glutathione to impart salt tolerance in *D. salina.* Chloroplastic 2-cys peroxiredoxin is an inevitable member of the photosynthetic antioxidant network, which was highly upregulated in high salt conditions. Observing the upraised antioxidant enzymes, both the organelles’ mitochondria and chloroplast accommodate an efficient antioxidant network to minimize the deleterious effect of excess accumulated ROS and to rebalance the redox state of the internal environment. Redox imbalance leads to oxidative damage of proteins, especially by the generation of disulfide bonds in the thiol group of cysteine residues which leads to the misfolding and loss of function of proteins. These oxidized proteins got repaired by a system of reductases and proteins, which provide reducing power to the reductases for their activity. Several transcripts show upregulation associated with repairing oxidized proteins, such as NADPH-dependent thioredoxin reductase (NTR), peptide methionine sulfoxide reductase (PMSR), thioredoxin-dependent peroxidase, thioredoxin-like protein. PMSR catalyzes the reduction of the methionine sulfoxide back to methionine residue in damaged proteins (Romero et al., 2004), showing a 2.2-fold upregulation in *D. salina* under salinity stress. The active participation of thioredoxins and thioredoxin-like proteins in thiol-disulfide interchange is well studied; these proteins activate the reductase activity of disulfide reductases. This system relies on ferredoxin as the reducing power; NADPH also acts as the source of reducing power for NTR-dependent redox regulation (Schürmann and Buchanan, 2008; Balsera and Buchanan, 2019). NTR dependent model revealed the active participation of 2-cys peroxiredoxins in modulating TRX-regulated enzymes (Awad et al., 2015).

ROS generation in the biological system is necessary for normal growth as well as for acclimation under different biotic and abiotic stress conditions. Excess ROS accumulation, such as singlet oxygen, hydrogen peroxide, superoxide, and hydroxyl radical accumulation, leads to the damage of biological molecules in the system (Mittler, 2017). To avoid this deleterious situation, a powerful ROS scavenging system and compartmentalization of ROS occur in organisms to balance the redox system. Chloroplast is a major site of ROS generation. The major sites that generate ROS include 1) the semiquinone radical generated at Cyt b_6_ complex transfer e- to O_2_ (Shochat et al., 1990), 2) ferredoxin can directly transfer an electron to O_2_ (Morehouse and Mason, 1988), 3) electron donation can happen from Cytb 559 cofactor of PSII to O_2_, 4) from reduced plastoquinone pool, 5) incomplete oxidation of H_2_O in water oxidation complex, 6) at PSI iron-sulfur clusters and phylloquinone donates an electron to O_2_, 7) presence of metal centers such as Mn, Fe convert H_2_O_2_ into hydroxyl radical (Macpherson et al., 1993). An imbalance in the redox state between PSI and PSII leads to the overexcitation of electrons in the apparatus, which turns on ROS generation. As per previous studies on ROS generation in photosynthetic apparatus, PSI generates H_2_O_2_ as well as O_2_^-^ and PSII generates O_2_^-^ alone as signaling molecules and makes the chloroplast an environmental sensor for initiating acclimation mechanisms at transcriptional as well as in the post-translational level (Asada, 1987).

There are reports stating cross-talk between ROS signaling and scavenging mechanisms with calcium signaling in cells under biotic and abiotic stress conditions. In plants, the intracellular calcium level-activated antioxidant system has been studied in several mutants, but the algal system is under-studied. Increased calmodulin (CAD) (TRINITY_DN6782_c0_g1_i12), calcium-dependent kinase (CDK) (TRINITY_DN4313_c0_g1_i45), calcium load activated calcium channel (TRINITY_DN878_c0_g2_i8), calcium-sensing protein (TRINITY_DN9636_c0_g1_i11), transcript accumulation brings the idea of intracellular elevated calcium level in *D. salina* under hypersalinity. Calmodulin is a ubiquitous calcium-sensing protein that functions as a signaling molecule in several physiological mechanisms, including abiotic stress tolerance in plants showing upregulation of 11-fold change in transcript data. The calcium load-activated calcium channel showed a 1.9-fold change, which usually helps release calcium ions from the endoplasmic reticulum to avoid overfilling. It shows the cells’ highly elevated calcium level under long-term hypersalinity conditions. CDPKs are an inevitable member of calcium-mediated signaling mechanisms to undertake several downstream processes, also offering moderate upregulation. ROS molecules are essential as signaling molecules, but their homeostasis is essential for the wellness of the intracellular environment. Calcium-calmodulin signaling plays an irreplaceable role in this regard. *In vitro* regulation of CAT3 expression by calcium-calmodulin signaling has been unveiled (Yang and Poovaiah, 2002). There are reports of peroxisomal Ca^2+^ mediated activation of enzymatic and non-enzymatic ROS scavenging mechanisms which control H_2_O_2_ levels (Costa et al., 2010). Also, in *A. thaliana*, calcium level elevation, and associated signaling mechanisms play a significant role in drought and salinity stress conditions (Knight et al., 1997). In *U. compressa*, marine algae, the activation of GR and GST is triggered by an elevation in both intracellular calcium levels and ROS (Gonzalez et al., 2010). Detailed analysis in Blast2GO of transcriptome shows many hypothetical and non-annotated transcripts with calcium-binding domains with significant fold change values; this clearly shows the indubitable calcium signaling mechanism in *D. salina*, and exhaustive studies in *D. salina* can lead to the revealing of new members and pathways in calcium signaling in salt tolerance.

### Retrograde signaling molecules involved in salinity tolerance

Research on chloroplast redox signaling molecules is limited in algal systems compared to that of plants, especially under abiotic stress tolerance. From the transcriptome data, we have fetched out the retrograde signaling molecules homologous to higher plants and *C. reinhardtii*, which showed significant upregulation in correlation with the ROS and calcium signaling molecule. Our transcriptome data show significant upregulation of most tetrapyrrole biosynthetic pathway enzymes (**Figures 6B, E**). But pigment analysis also showed decreased chlorophyll molecule (**Supplementary Figure 5**). So there is a possibility of inhibition of this pathway and accumulation of TPB pathway intermediates. An interesting chloroplastic transcript showing highly significant upregulation was s-FLP, an alternative splicing variant of the FLU-like gene (FLP). In *C. reinhardtii* there is a report of two transcripts of FLU-like gene (FLP) formed by alternative splicing, s-FLP, and l-FLP variants. Also, the alternative splicing of FLP has been reported as a blue light-induced mechanism, and mutant studies revealed chlorophyll biosynthesis intermediates PROTO-IX, Mg-PROTO-IX, and P-Chlide regulate the level of s-FLP and l-FLP (Falciatore et al., 2005).

**Figure 9:**
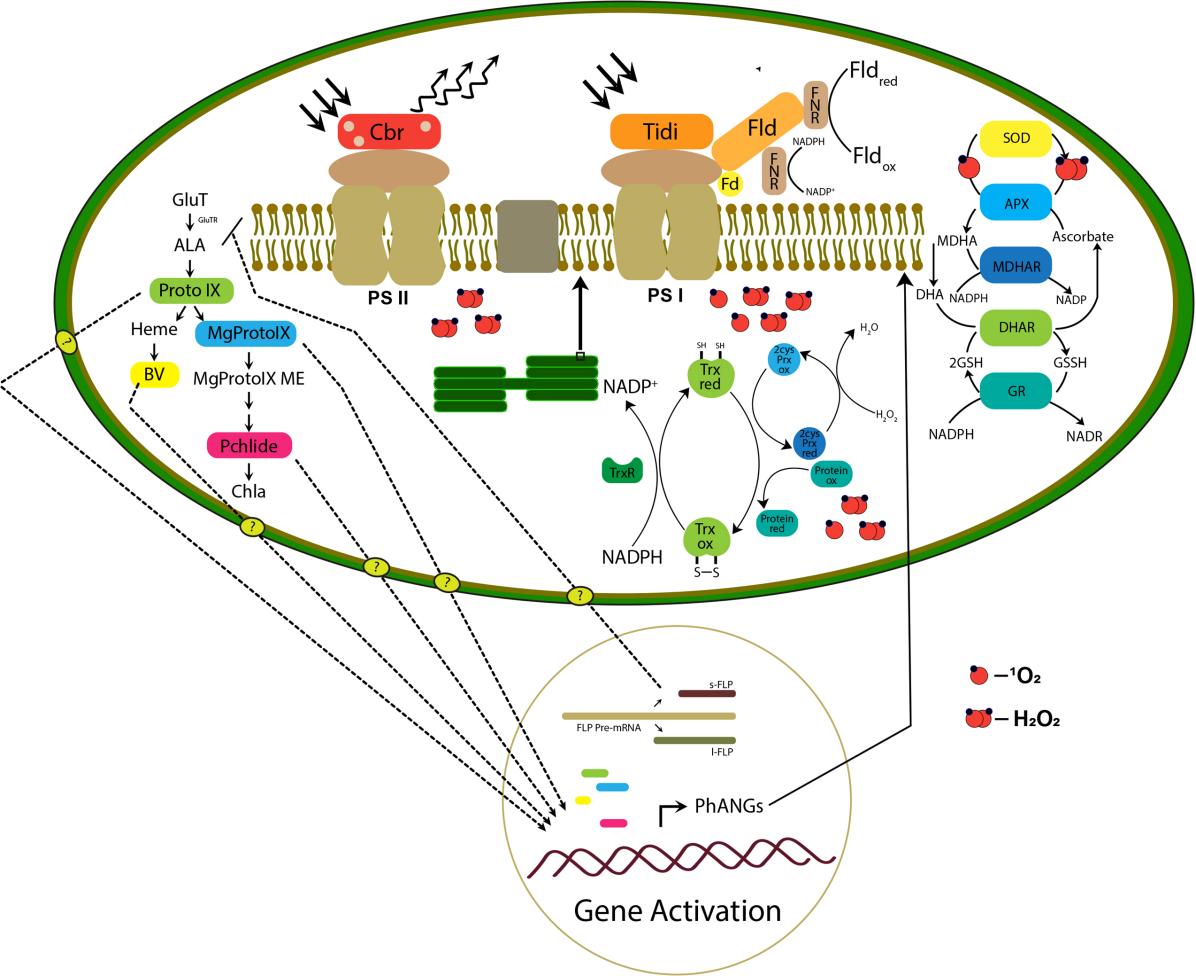
The hypothetical model proposed is based on the transcriptomic study representing the retrograde signaling and remodeling of photosynthetic apparatus in the chloroplast. The right panel of the figure represents the TPB pathway intermediate accumulation and retrograde signaling mechanism in which the transport of molecules to the nucleus is still unknown. The photosynthetic apparatus represents possible remodeling initiated by the photosystem-associated gene expression (PhANGs) with the involvement of chloroplast Tidi, CBR, and flavodoxin IsiB. Also, the efficient chloroplast ROS scavenging and protein repair mechanism is represented.

Further studies have proven the protective role of these splicing variants against oxidative damage under varying light conditions by acting as retrograde signals from the plastid to the nucleus. Among these unique forms, s-FLP shows an upregulation of 8-fold change under hypersalinity conditions. It strongly indicates an FLP variant-mediated complex retrograde regulatory mechanism acting on chloroplast to acclimate under salt stress. The GUN5 mutant study revealed the function of the Chl H subunit of Mg-chelatase in inserting the Mg^2+^ into protoporphyrin IX in chlorophyll biosynthesis (Mochizuki et al., 2001) and also showed a moderate upregulation. Significant upregulation was also observed in heme oxygenase, mediates the cleavage of heme molecules under high salt. Oxidative breakdown of heme generates bilin molecules in algae, and they lack photosensors such as phytochrome. In Chlamydomonas, a bilin molecule-mediated retrograde signaling mechanism has been unveiled, which induces PhANGs expression as photoprotection as well as efficiently triggers metabolic pathways for the detoxification of ROS molecules (Duanmu et al., 2013). In our case, we expect bilin molecules as strong retrograde signals in PhANGs expression and the ROS detoxification system (**Figure 9**).

## Conclusion

*D. salina*, as highly salt-tolerant green algae that grow in extremely saline environments, exhibits efficient acclimation mechanisms, especially at the chloroplastic level. Our transcriptomic analysis of chloroplastic genes and experimental approach support an establishment of efficient chloroplast retrograde signaling and possible remodeling of photosynthetic apparatus in *D. salina* under long-term hypersaline conditions. The direct and indirect effect of hypersaline conditions imposes a robust retrograde signaling mechanism by a possible cross-talk between the ROS and calcium signaling mechanism. The transcripts involved in both mechanisms show an uptrend pattern and positive correlation. Chloroplast acts as a primary environmental sensor. ROS accumulation in chloroplast was observed and the accumulated ROS leads to proportionate damage to photosystem proteins. The transcript analysis has proposed an efficient PSII repair and assembly mechanism such as Psb27, Psb28, PsbZ PsbT, and PsbM photosystem subunits. The major two transcripts upregulated in the PSI system chloroplast Tidi and flavodoxin IsiB, tempting to suggest a possible remodeling at a super complex level to rebalance the energy distribution and electron transport in the apparatus. The expression of CBR protein which was earlier reported in *D. bardawil*, having a photoprotection function in high light showed a correlation with our carotenoid biosynthesis transcripts upregulation; this suggests a protective role of CBR under the hypersaline condition to the PS II supercomplex. Expression pattern of ROS scavenging associated and protein refolding related transcripts such as Glutathione S-transferase, glutathione reductase, Chloroplastic 2-cys peroxiredoxin, NADPH-dependent thioredoxin reductase (NTR), peptide methionine sulfoxide reductase (PMSR), thioredoxin-dependent peroxidase, thioredoxin-like protein supports an efficient retrograde signaling mechanism. Tetrapyrrole biosynthesis pathway intermediates such as PROTO-IX, Mg-PROTO-IX, and P-Chlide are reported to act as retrograde signals in several studies. Also, the oxidation product of heme, the biliverdin molecules, suggested acting as retrograde signals. Our transcript analysis of TPB pathway enzymes and expression of the splicing variant of s-FLP, which was earlier reported as a negative regulator of the TPB pathway, supports the contribution of the TPB pathway in possible retrograde signaling mechanism and PhANGs expression. All these mechanisms play synergistically to rebalance the photosynthetic apparatus’s redox state and help cope with the hypersaline environment in *D. salina.* We hope that the genes expressed in *D. salina* to the hypersaline condition could be transferred to the land plants/fresh algae to withstand the high salinity conditions and improve crop yield.

## Data availability statement

The datasets presented in this study can be found in online repositories. The names of the repository/repositories and accession number(s) can be found in the article/**Supplementary Material**.

## Author contributions

RS procured the grants and designed the experiments. PR performed most of the experiments while RY, PS and AK did some experiments. PR and NP together analyzed the transcriptome data. RS and PR analyzed the results and wrote the first draft. All authors contributed to the article and approved the submitted version.

## Supplementary material

Supplementary Figure 1 **(A)** Principal component analysis of transcript data obtained from control and hypersaline condition to see how replicates within the sample cluster together in one group and how samples differ in two different conditions. **(B)** sample correlation among the samples and within the replicates represented in heatmap form.

Supplementary Figure 2 Differentially expressed gene expression pattern. **(A)** A volcano plot of differentially expressed genes was generated among the mapped genes using the Dseq2 program with a fold change of ≥ 0.5 and FDR ≤ 0.05. **(B)** Heat map generated from the differentially expressed genes from the triplicates of each sample.

Supplementary Figure 3 Real-time PCR analysis of 10 significant differentially expressed transcripts selected. RPL2 ribosomal protein was selected as the internal control. Each gene was tested with three biological replicates and was calculated.

Supplementary Figure 4 Growth kinetics and biomass measurement. **(A)** Cell density and **(B)** cell number of D. salina cells grown under control, 2M, and 3M conditions were analyzed throughout 24 hrs. **(C)** Morphological observation of change in color of culture from green (control), yellowish-green (hypersaline). **(D)** Biomass obtained after 72 hrs. of growth under control, 2M, and 3M conditions were quantified after lyophilization for 24 hrs. Statistical analysis was done using one-way ANOVA (Bonferroni t test) was performed to measure the P-values (ns, not significant). Asterisks indicate the level of significance of the drought effect compared to the respective control: ***P < 0.001.

Supplementary Figure 5 The chlorophyll and carotenoid pigment content in control and treated samples were measured per 1 ml of cells after 73 h of growth. UV-Visible spectrophotometer measurements in different wavelengths 480, 520, 645, and 663. The concentration was measured using the equations described in Lichtenthal (Lichtenthaler, 1987) and Porra et al. (1989).

Supplementary Table 1 Primers designed for real-time PCR analysis of 10 significant differentially expressed transcripts.

Supplementary Table 2 List of transcripts in **(A)** Photosystem-associated transcripts, **(B)** ROS and calcium signaling associated transcripts, **(C)** Carotenoid biosynthesis associated pathway, along with trinity IDs, log2 fold change and FPKM values.
