# Supplementary material for: Photosynthetic efficiency and transcriptome analysis of Dunaliella salina under hypersaline: a retrograde signaling mechanism in the chloroplast
Source: Front Plant Sci. 2023 Jun 21;14:1192258. doi: 10.3389/fpls.2023.1192258 (PMC10322210; doi:10.3389/fpls.2023.1192258)
Supplement: Supplementary Figure 1 — (A) Principal component analysis of transcript data obtained from control and hypersaline condition to see how replicates within the sample cluster together in one group and how samples differ in two different conditions. (B) sample correlation among the samples and within the replicates represented in heatmap form. [file Presentation_1.pptx]

## Slide 1
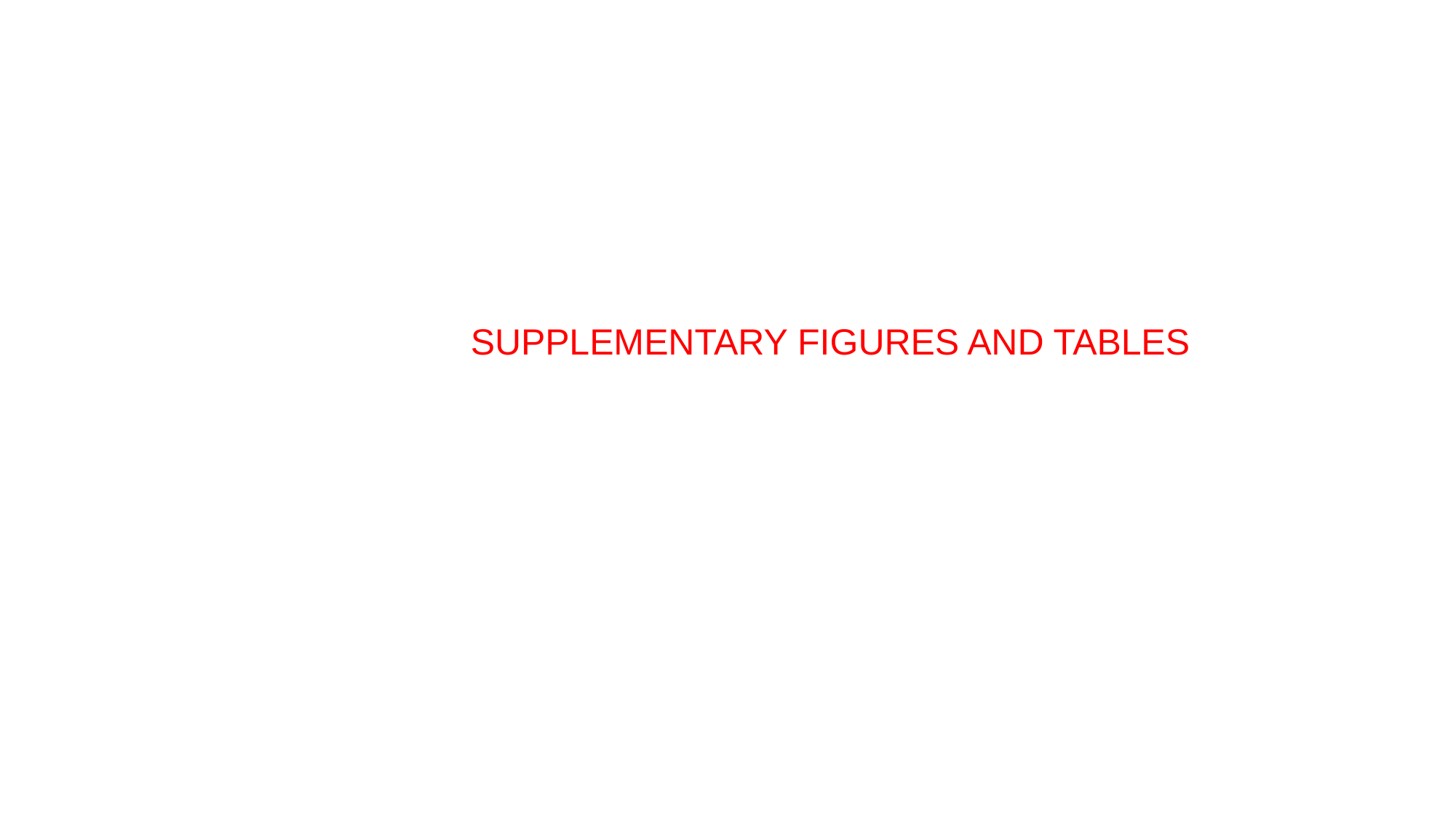

SUPPLEMENTARY FIGURES AND TABLES

## Slide 2
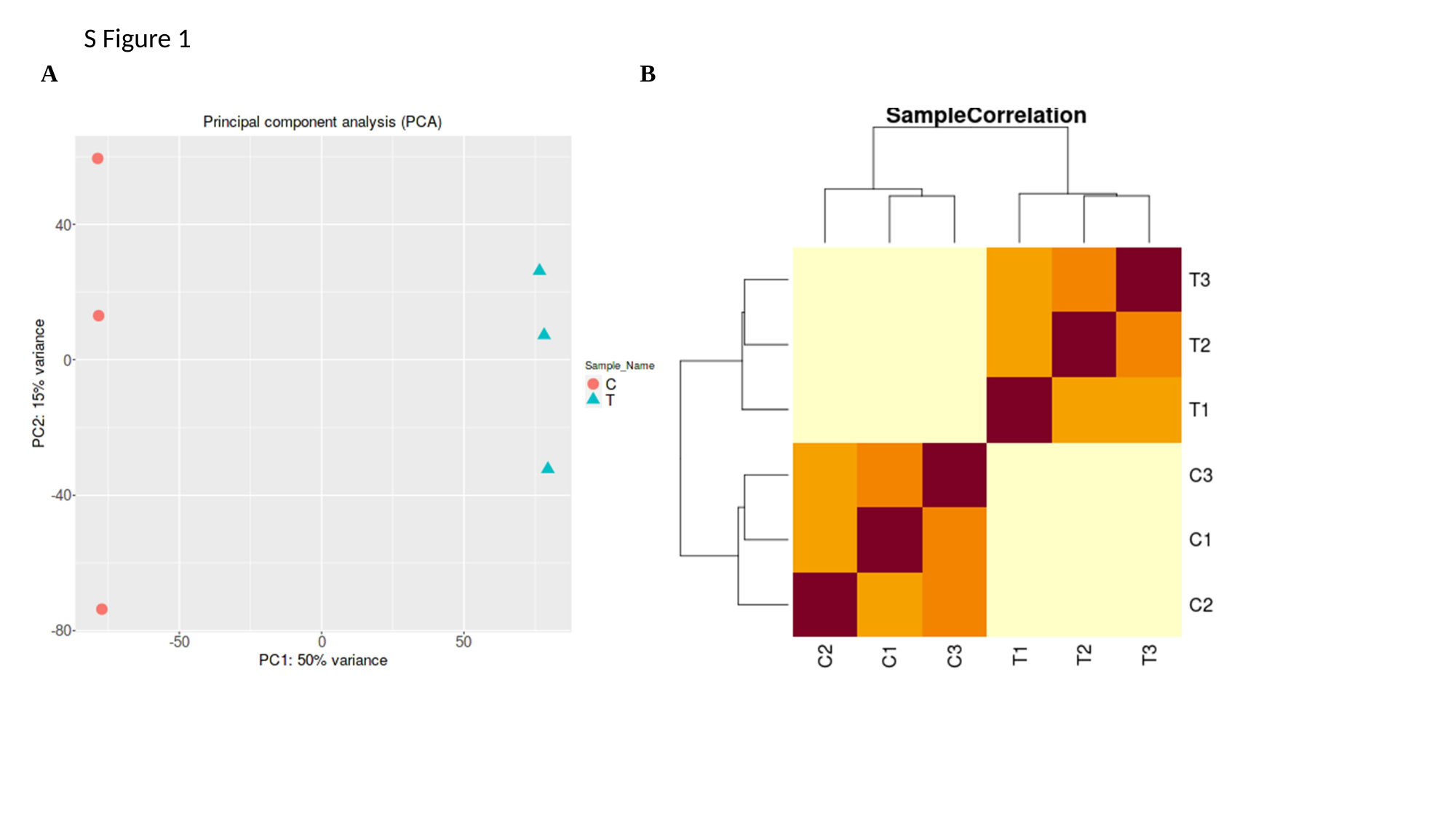

S Figure 1
A
B

## Slide 3
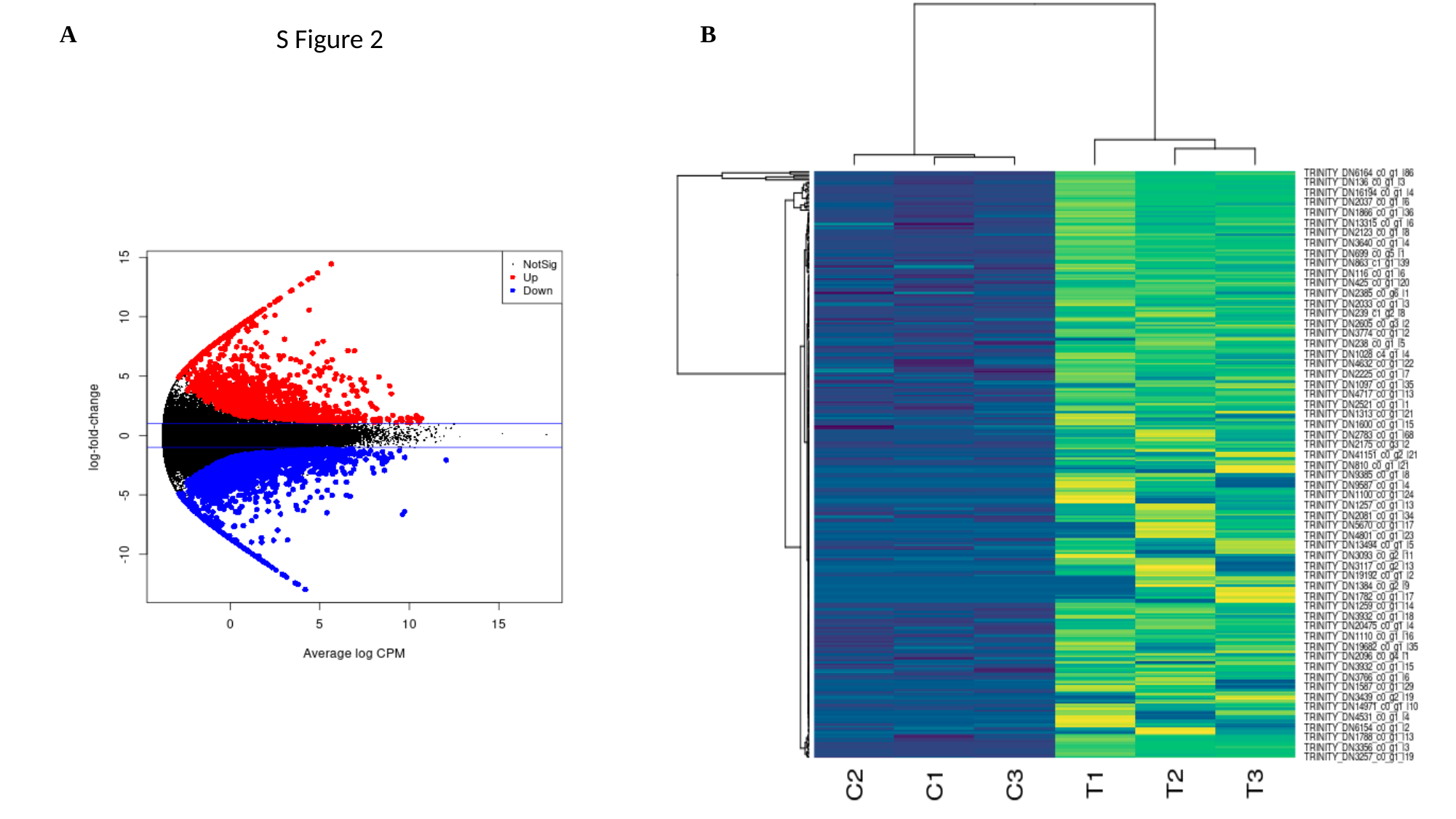

A
B
S Figure 2

## Slide 4
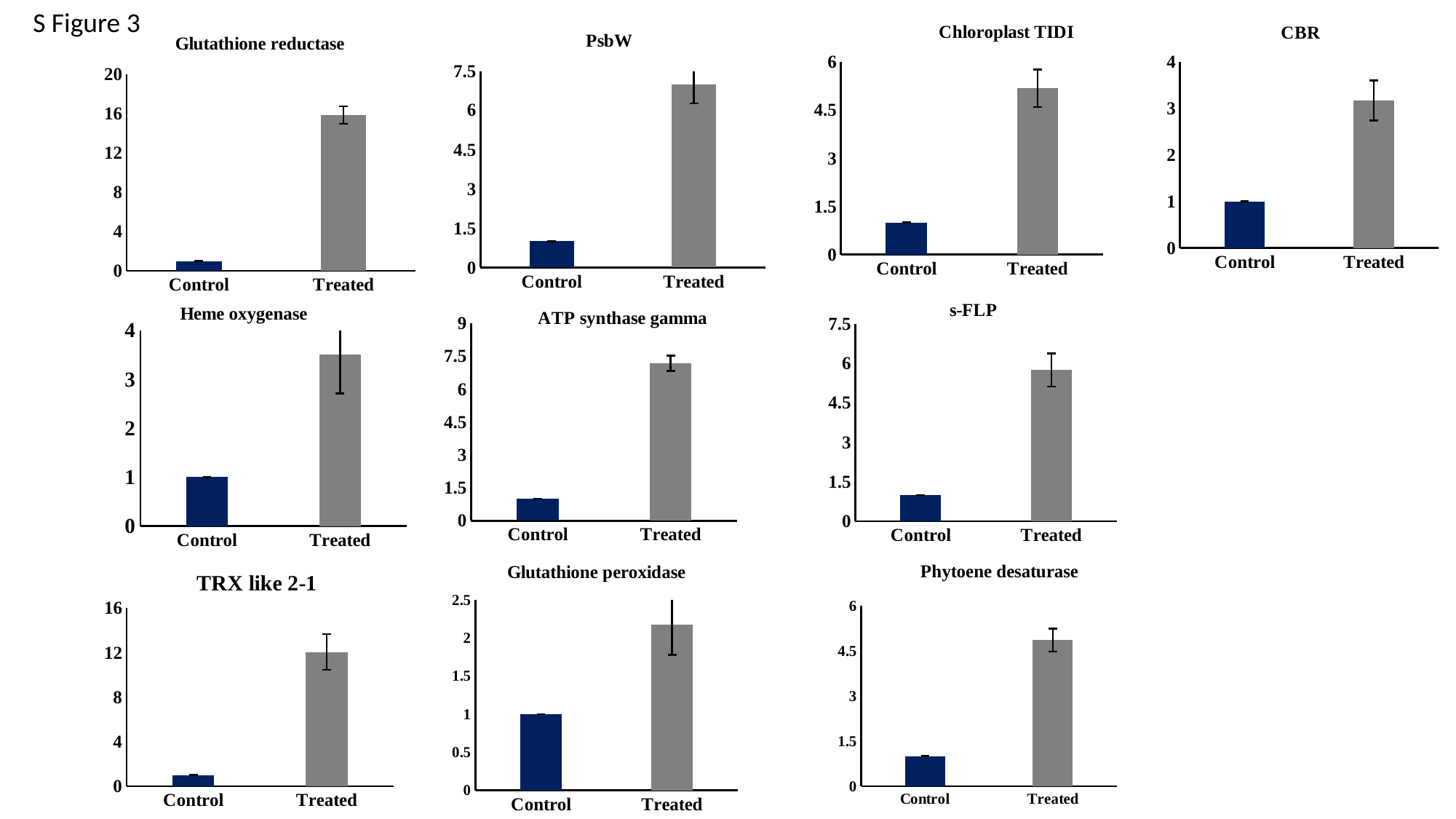

S Figure 3
### Chart:
| Category | Glutathione reductase |
|---|---|
| Control | 1.0 |
| Treated | 15.85137119356529 |
### Chart:
| Category | Chloroplast TIDI |
|---|---|
| Control | 1.0 |
| Treated | 5.183618908037819 |
### Chart:
| Category | CBR |
|---|---|
| Control | 1.0 |
| Treated | 3.1743491172758804 |
### Chart:
| Category | PsbW |
|---|---|
| Control | 1.0 |
| Treated | 6.999014562628402 |
### Chart:
| Category | ATP synthase gamma |
|---|---|
| Control | 1.0 |
| Treated | 7.182612070374983 |
### Chart:
| Category | s-FLP |
|---|---|
| Control | 1.0 |
| Treated | 5.746249586624304 |
### Chart:
| Category | Heme oxygenase |
|---|---|
| Control | 1.0 |
| Treated | 3.5053144176201165 |
### Chart:
| Category | Glutathione peroxidase |
|---|---|
| Control | 1.0 |
| Treated | 2.180733502729259 |
### Chart: Phytoene desaturase
| Category | |
|---|---|
| Control | 1.0 |
| Treated | 4.868148218139969 |
### Chart: TRX like 2-1
| Category | TRX LIKE 2-1 |
|---|---|
| Control | 1.0 |
| Treated | 12.043142124310862 |

## Slide 5
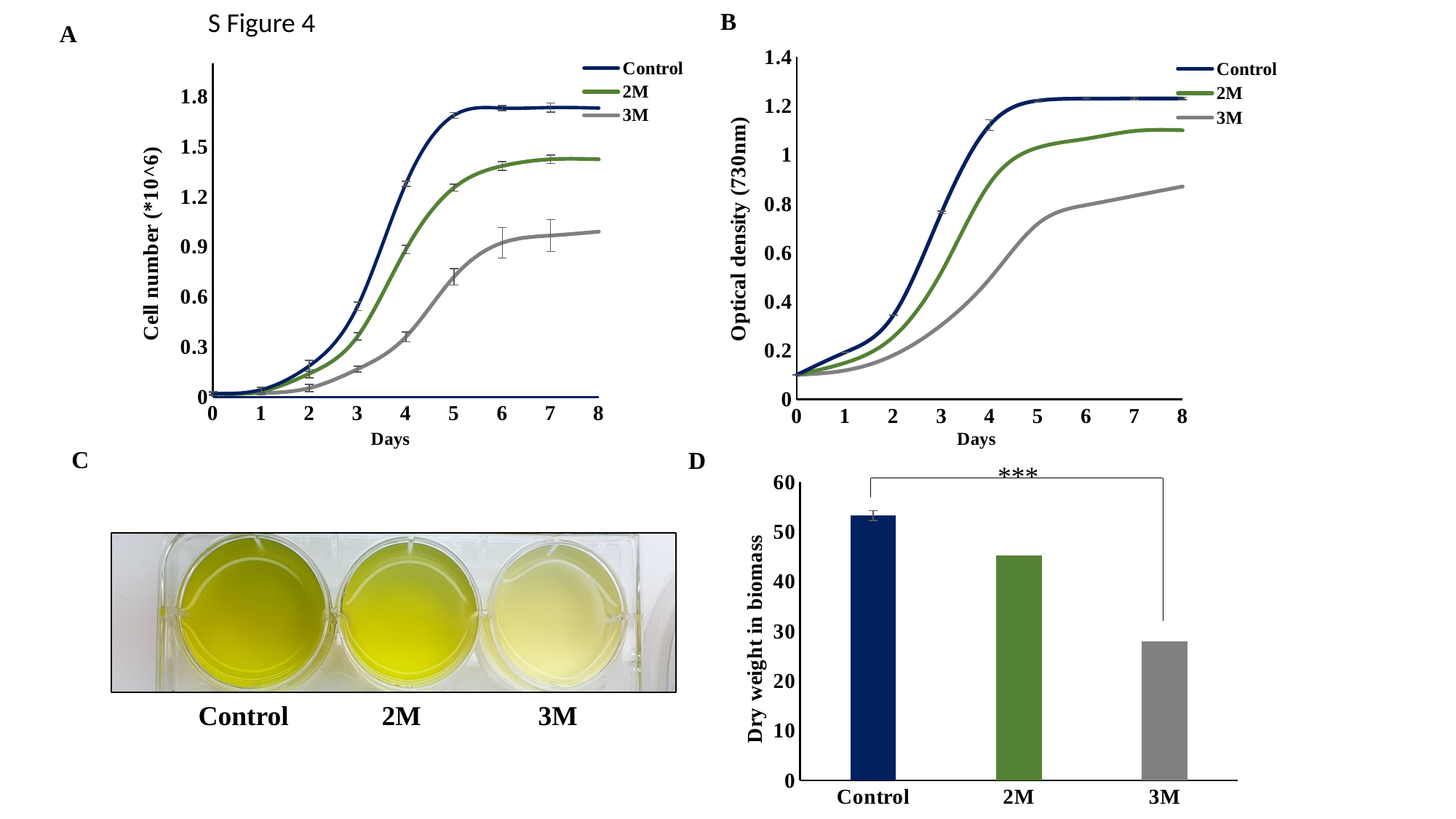

S Figure 4
B
A
### Chart
| Category | Control | 2M | 3M |
|---|---|---|---|
### Chart
| Category | Control | 2M | 3M |
|---|---|---|---|
### Chart
| Category | Dry weight in biomass(mg/L) |
|---|---|
| Control | 53.28516666666667 |
| 2M | 45.300000000000004 |
| 3M | 27.96666666666667 |Control
2M
3M
C
D
***

## Slide 6
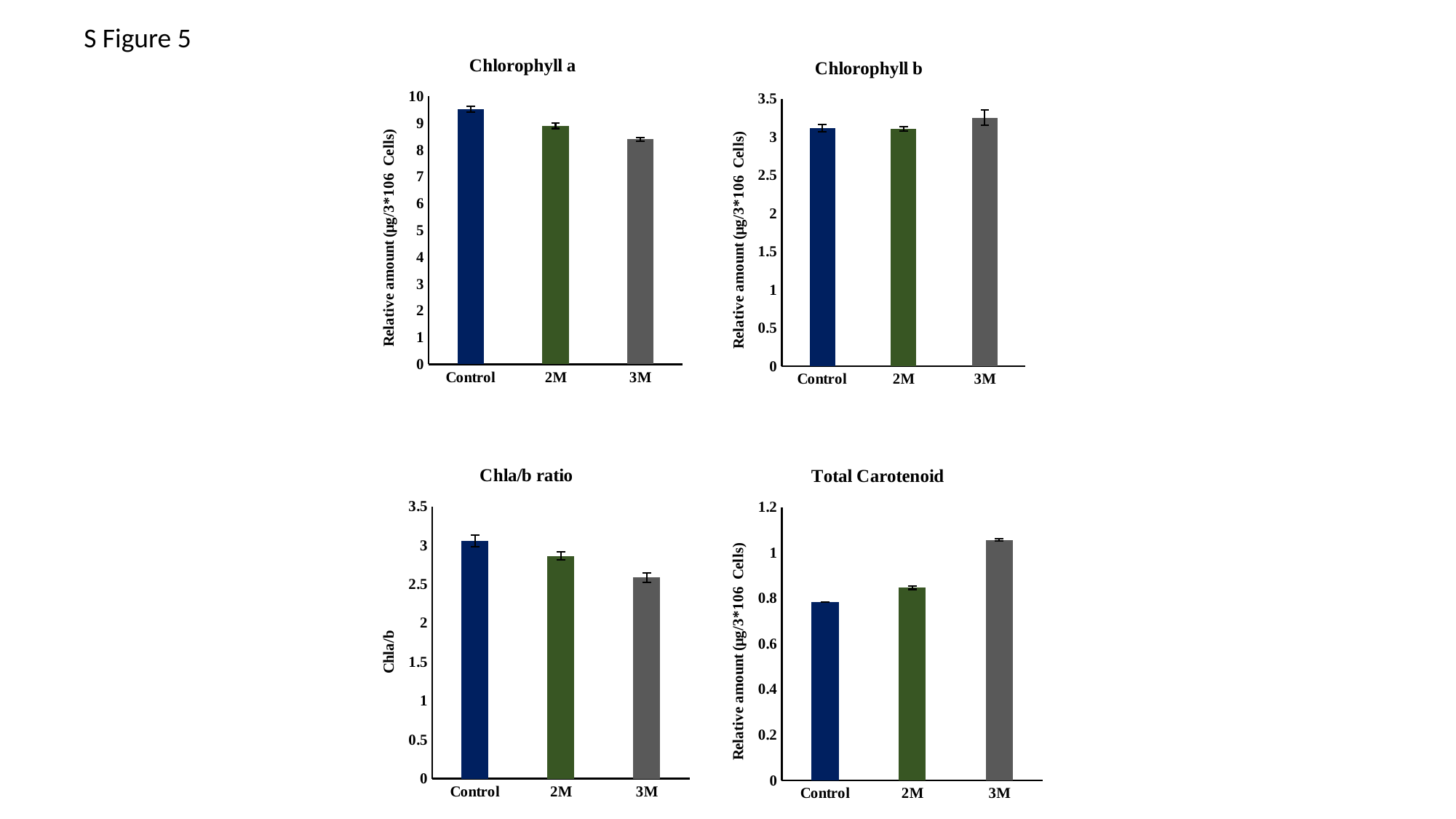

S Figure 5
### Chart: Chlorophyll a
| Category | |
|---|---|
| Control | 9.520289999999997 |
| 2M | 8.901283333333334 |
| 3M | 8.398663333333332 |
### Chart: Chlorophyll b
| Category | |
|---|---|
| Control | 3.1154733333333327 |
| 2M | 3.1098866666666662 |
| 3M | 3.2512866666666658 |
### Chart: Chla/b ratio
| Category | |
|---|---|
| Control | 3.056986541480852 |
| 2M | 2.862615198064925 |
| 3M | 2.5850056121538807 |
### Chart: Total Carotenoid
| Category | |
|---|---|
| Control | 0.7838693333333334 |
| 2M | 0.847194 |
| 3M | 1.0565766666666667 |

## Slide 7
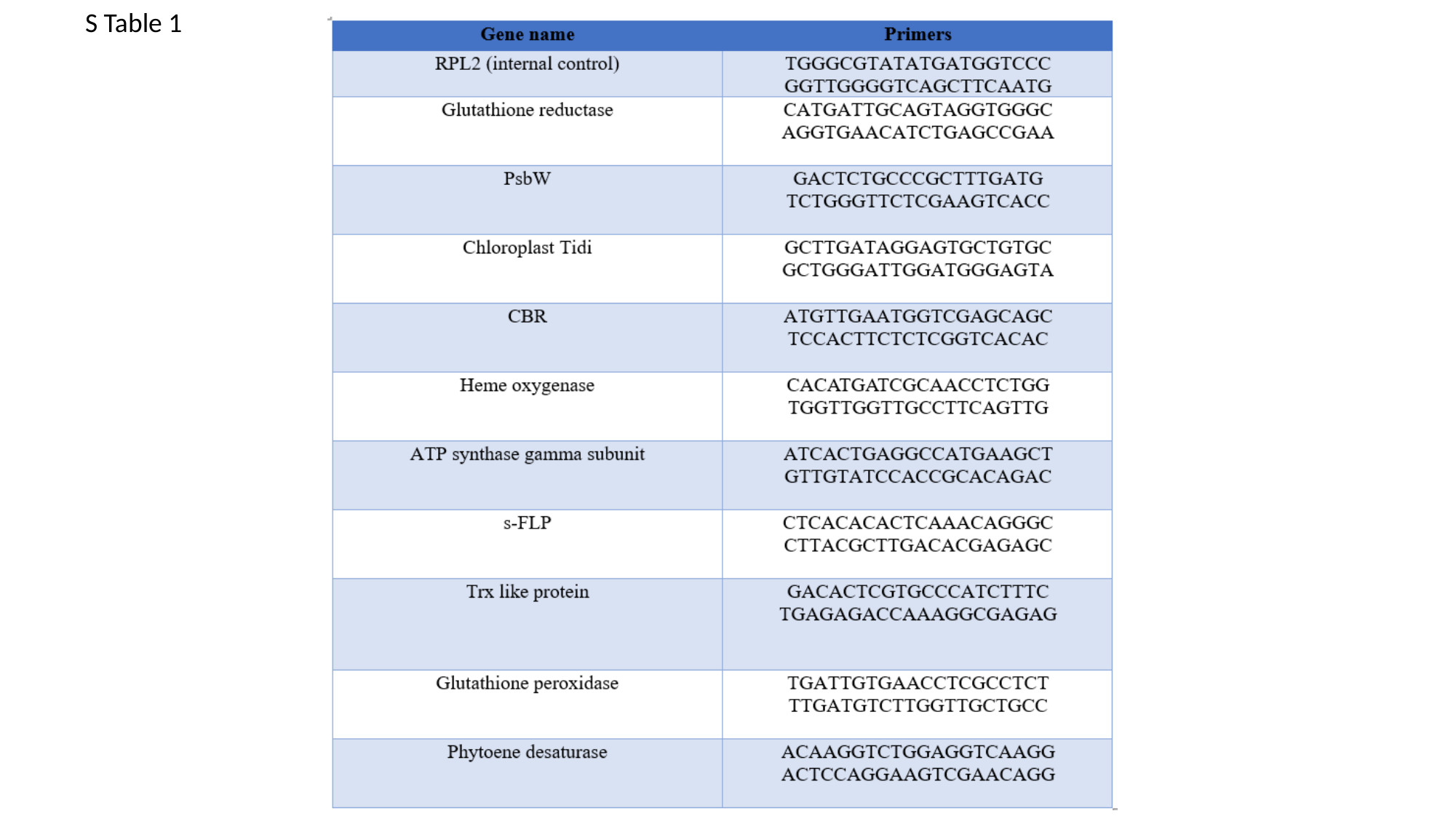

S Table 1

## Slide 8
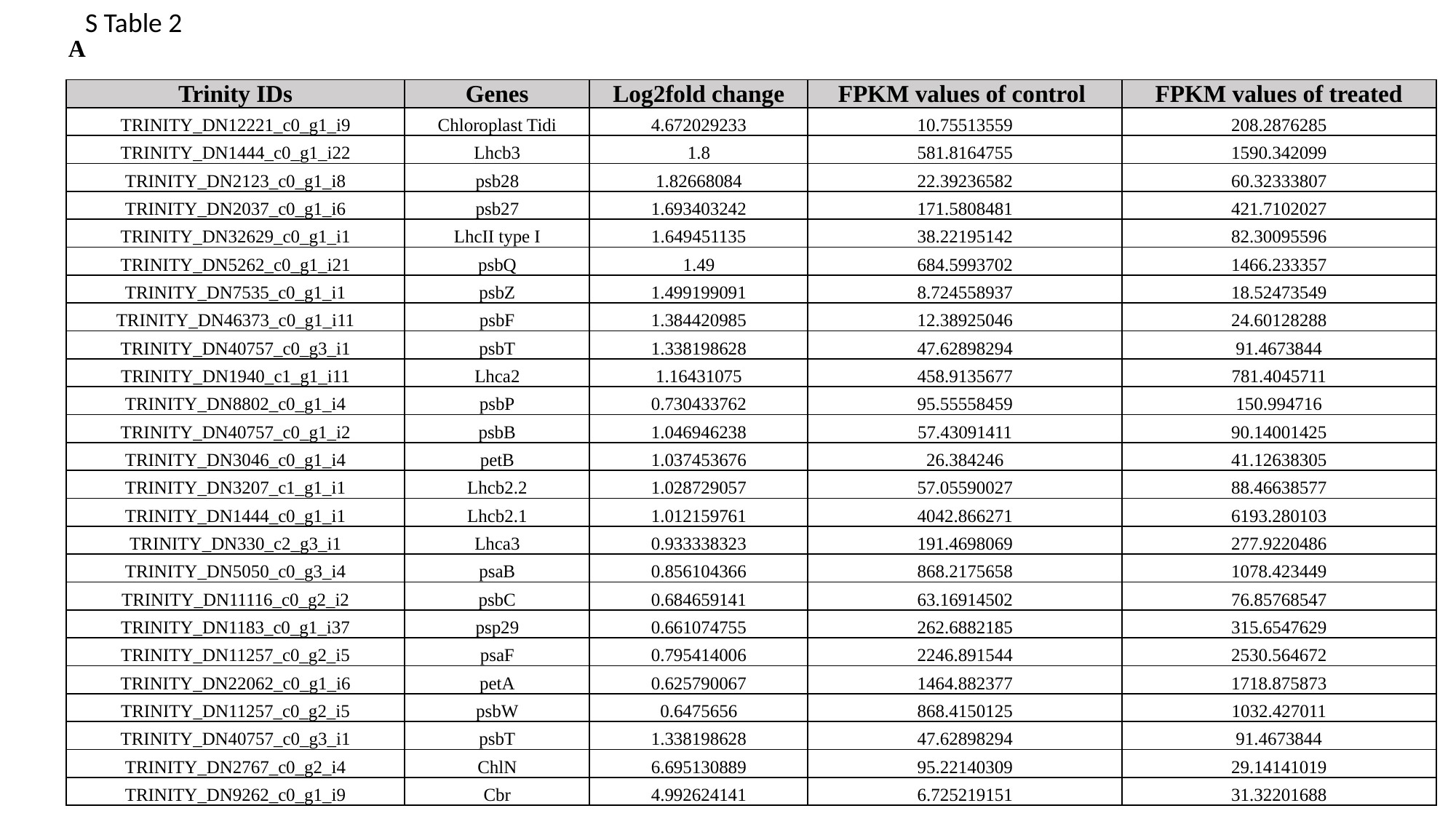

S Table 2
A
| Trinity IDs | Genes | Log2fold change | FPKM values of control | FPKM values of treated |
| --- | --- | --- | --- | --- |
| TRINITY\_DN12221\_c0\_g1\_i9 | Chloroplast Tidi | 4.672029233 | 10.75513559 | 208.2876285 |
| TRINITY\_DN1444\_c0\_g1\_i22 | Lhcb3 | 1.8 | 581.8164755 | 1590.342099 |
| TRINITY\_DN2123\_c0\_g1\_i8 | psb28 | 1.82668084 | 22.39236582 | 60.32333807 |
| TRINITY\_DN2037\_c0\_g1\_i6 | psb27 | 1.693403242 | 171.5808481 | 421.7102027 |
| TRINITY\_DN32629\_c0\_g1\_i1 | LhcII type I | 1.649451135 | 38.22195142 | 82.30095596 |
| TRINITY\_DN5262\_c0\_g1\_i21 | psbQ | 1.49 | 684.5993702 | 1466.233357 |
| TRINITY\_DN7535\_c0\_g1\_i1 | psbZ | 1.499199091 | 8.724558937 | 18.52473549 |
| TRINITY\_DN46373\_c0\_g1\_i11 | psbF | 1.384420985 | 12.38925046 | 24.60128288 |
| TRINITY\_DN40757\_c0\_g3\_i1 | psbT | 1.338198628 | 47.62898294 | 91.4673844 |
| TRINITY\_DN1940\_c1\_g1\_i11 | Lhca2 | 1.16431075 | 458.9135677 | 781.4045711 |
| TRINITY\_DN8802\_c0\_g1\_i4 | psbP | 0.730433762 | 95.55558459 | 150.994716 |
| TRINITY\_DN40757\_c0\_g1\_i2 | psbB | 1.046946238 | 57.43091411 | 90.14001425 |
| TRINITY\_DN3046\_c0\_g1\_i4 | petB | 1.037453676 | 26.384246 | 41.12638305 |
| TRINITY\_DN3207\_c1\_g1\_i1 | Lhcb2.2 | 1.028729057 | 57.05590027 | 88.46638577 |
| TRINITY\_DN1444\_c0\_g1\_i1 | Lhcb2.1 | 1.012159761 | 4042.866271 | 6193.280103 |
| TRINITY\_DN330\_c2\_g3\_i1 | Lhca3 | 0.933338323 | 191.4698069 | 277.9220486 |
| TRINITY\_DN5050\_c0\_g3\_i4 | psaB | 0.856104366 | 868.2175658 | 1078.423449 |
| TRINITY\_DN11116\_c0\_g2\_i2 | psbC | 0.684659141 | 63.16914502 | 76.85768547 |
| TRINITY\_DN1183\_c0\_g1\_i37 | psp29 | 0.661074755 | 262.6882185 | 315.6547629 |
| TRINITY\_DN11257\_c0\_g2\_i5 | psaF | 0.795414006 | 2246.891544 | 2530.564672 |
| TRINITY\_DN22062\_c0\_g1\_i6 | petA | 0.625790067 | 1464.882377 | 1718.875873 |
| TRINITY\_DN11257\_c0\_g2\_i5 | psbW | 0.6475656 | 868.4150125 | 1032.427011 |
| TRINITY\_DN40757\_c0\_g3\_i1 | psbT | 1.338198628 | 47.62898294 | 91.4673844 |
| TRINITY\_DN2767\_c0\_g2\_i4 | ChlN | 6.695130889 | 95.22140309 | 29.14141019 |
| TRINITY\_DN9262\_c0\_g1\_i9 | Cbr | 4.992624141 | 6.725219151 | 31.32201688 |

## Slide 9
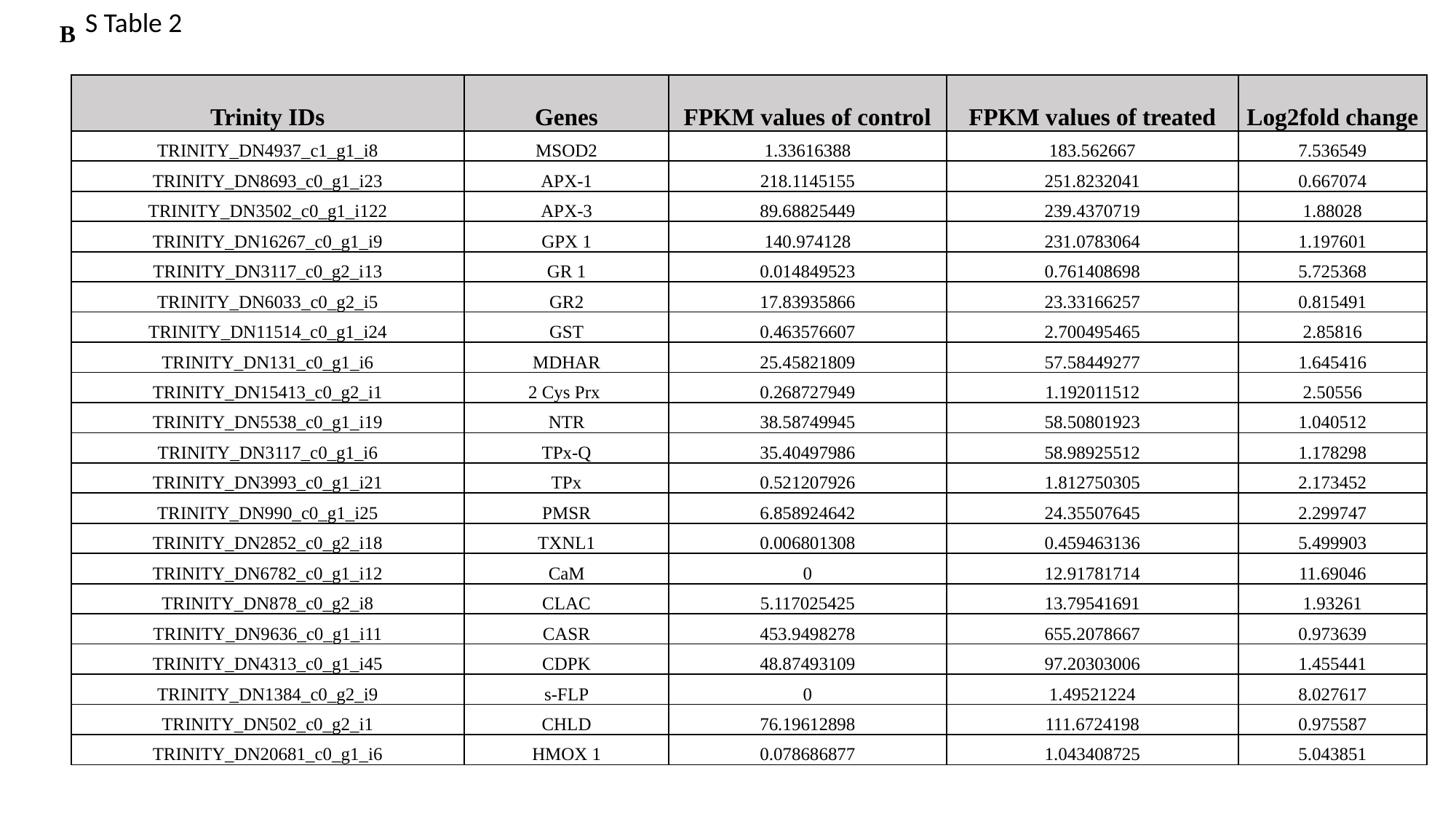

S Table 2
B
| Trinity IDs | Genes | FPKM values of control | FPKM values of treated | Log2fold change |
| --- | --- | --- | --- | --- |
| TRINITY\_DN4937\_c1\_g1\_i8 | MSOD2 | 1.33616388 | 183.562667 | 7.536549 |
| TRINITY\_DN8693\_c0\_g1\_i23 | APX-1 | 218.1145155 | 251.8232041 | 0.667074 |
| TRINITY\_DN3502\_c0\_g1\_i122 | APX-3 | 89.68825449 | 239.4370719 | 1.88028 |
| TRINITY\_DN16267\_c0\_g1\_i9 | GPX 1 | 140.974128 | 231.0783064 | 1.197601 |
| TRINITY\_DN3117\_c0\_g2\_i13 | GR 1 | 0.014849523 | 0.761408698 | 5.725368 |
| TRINITY\_DN6033\_c0\_g2\_i5 | GR2 | 17.83935866 | 23.33166257 | 0.815491 |
| TRINITY\_DN11514\_c0\_g1\_i24 | GST | 0.463576607 | 2.700495465 | 2.85816 |
| TRINITY\_DN131\_c0\_g1\_i6 | MDHAR | 25.45821809 | 57.58449277 | 1.645416 |
| TRINITY\_DN15413\_c0\_g2\_i1 | 2 Cys Prx | 0.268727949 | 1.192011512 | 2.50556 |
| TRINITY\_DN5538\_c0\_g1\_i19 | NTR | 38.58749945 | 58.50801923 | 1.040512 |
| TRINITY\_DN3117\_c0\_g1\_i6 | TPx-Q | 35.40497986 | 58.98925512 | 1.178298 |
| TRINITY\_DN3993\_c0\_g1\_i21 | TPx | 0.521207926 | 1.812750305 | 2.173452 |
| TRINITY\_DN990\_c0\_g1\_i25 | PMSR | 6.858924642 | 24.35507645 | 2.299747 |
| TRINITY\_DN2852\_c0\_g2\_i18 | TXNL1 | 0.006801308 | 0.459463136 | 5.499903 |
| TRINITY\_DN6782\_c0\_g1\_i12 | CaM | 0 | 12.91781714 | 11.69046 |
| TRINITY\_DN878\_c0\_g2\_i8 | CLAC | 5.117025425 | 13.79541691 | 1.93261 |
| TRINITY\_DN9636\_c0\_g1\_i11 | CASR | 453.9498278 | 655.2078667 | 0.973639 |
| TRINITY\_DN4313\_c0\_g1\_i45 | CDPK | 48.87493109 | 97.20303006 | 1.455441 |
| TRINITY\_DN1384\_c0\_g2\_i9 | s-FLP | 0 | 1.49521224 | 8.027617 |
| TRINITY\_DN502\_c0\_g2\_i1 | CHLD | 76.19612898 | 111.6724198 | 0.975587 |
| TRINITY\_DN20681\_c0\_g1\_i6 | HMOX 1 | 0.078686877 | 1.043408725 | 5.043851 |

## Slide 10
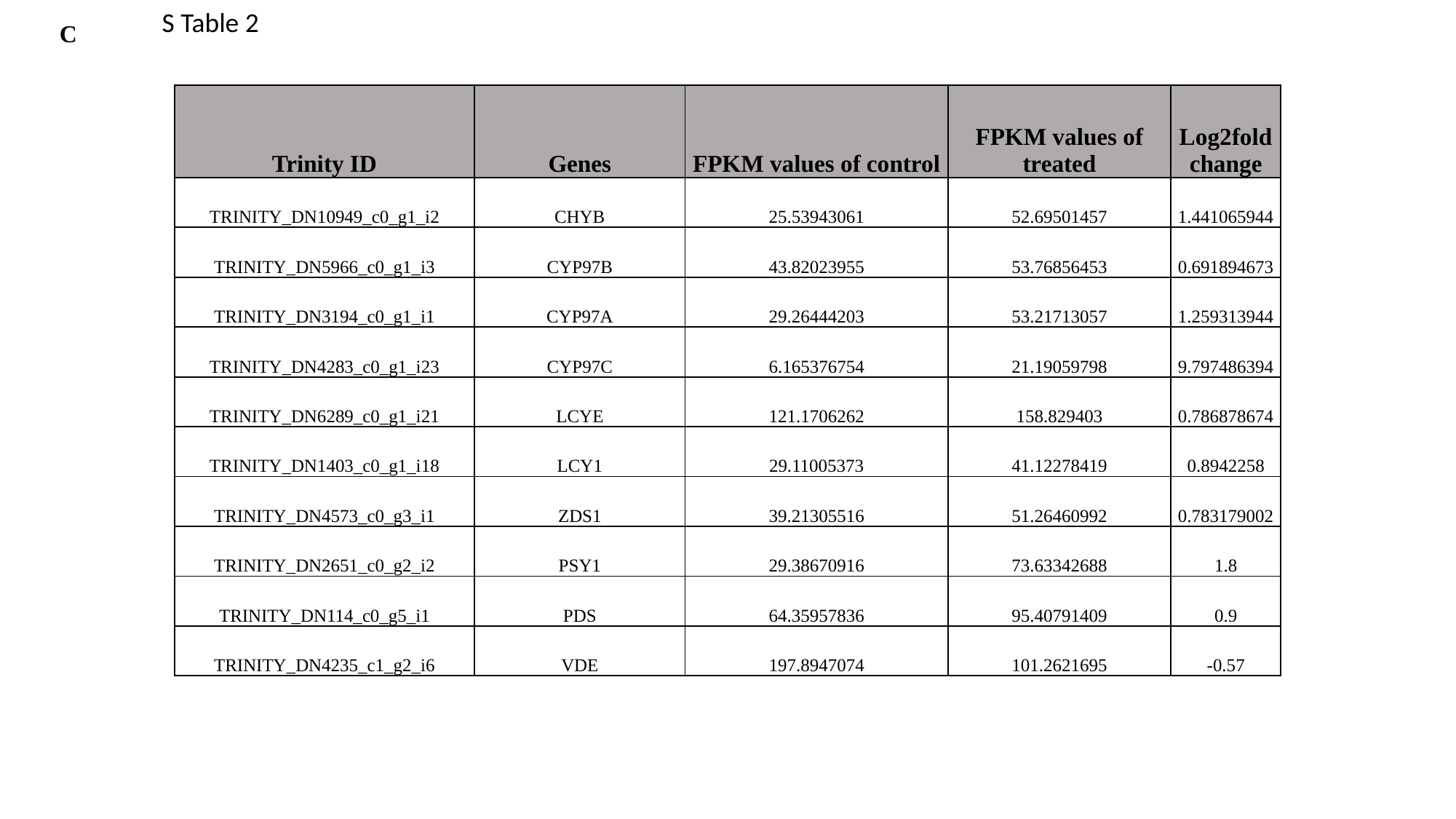

S Table 2
C
| Trinity ID | Genes | FPKM values of control | FPKM values of treated | Log2fold change |
| --- | --- | --- | --- | --- |
| TRINITY\_DN10949\_c0\_g1\_i2 | CHYB | 25.53943061 | 52.69501457 | 1.441065944 |
| TRINITY\_DN5966\_c0\_g1\_i3 | CYP97B | 43.82023955 | 53.76856453 | 0.691894673 |
| TRINITY\_DN3194\_c0\_g1\_i1 | CYP97A | 29.26444203 | 53.21713057 | 1.259313944 |
| TRINITY\_DN4283\_c0\_g1\_i23 | CYP97C | 6.165376754 | 21.19059798 | 9.797486394 |
| TRINITY\_DN6289\_c0\_g1\_i21 | LCYE | 121.1706262 | 158.829403 | 0.786878674 |
| TRINITY\_DN1403\_c0\_g1\_i18 | LCY1 | 29.11005373 | 41.12278419 | 0.8942258 |
| TRINITY\_DN4573\_c0\_g3\_i1 | ZDS1 | 39.21305516 | 51.26460992 | 0.783179002 |
| TRINITY\_DN2651\_c0\_g2\_i2 | PSY1 | 29.38670916 | 73.63342688 | 1.8 |
| TRINITY\_DN114\_c0\_g5\_i1 | PDS | 64.35957836 | 95.40791409 | 0.9 |
| TRINITY\_DN4235\_c1\_g2\_i6 | VDE | 197.8947074 | 101.2621695 | -0.57 |
